# Water-Soluble Fullerenol with Hydroxyl Group Dependence for Efficient Two-Photon Excited Photodynamic Inactivation of Infectious Microbes

**DOI:** 10.1186/s11671-020-03329-6

**Published:** 2020-05-06

**Authors:** Wen-Shuo Kuo, Jiu-Yao Wang, Chia-Yuan Chang, Jui-Chang Liu, Yu-Ting Shao, Yen-Sung Lin, Edmund Cheung So, Ping-Ching Wu

**Affiliations:** 1 School of Chemistry and Materials Science, Nanjing University of Information Science and Technology, Nanjing, 210044 Jiangsu China; 2 Allergy & Clinical Immunology Research Center, National Cheng Kung University Hospital, College of Medicine, National Cheng Kung University, Tainan, 701 Taiwan, Republic of China; 3 Department of Biochemistry and Molecular Biology, National Cheng Kung University Hospital, College of Medicine, National Cheng Kung University, Tainan, 701 Taiwan, Republic of China; 4 Department of Microbiology & Immunology, National Cheng Kung University Hospital, College of Medicine, National Cheng Kung University, Tainan, 701 Taiwan, Republic of China; 5 Department of Mechanical Engineering, National Cheng Kung University, Tainan, 701 Taiwan, Republic of China; 6 Division of Pulmonary and Critical Care Medicine, An Nan Hospital, China Medical University, Tainan, 709 Taiwan, Republic of China; 7 Department of Nursing, Chung Hwa University of Medical Technology, Tainan, 717 Taiwan, Republic of China; 8 Department of Anesthesia & Medicine Research, An Nan Hospital, China Medical University, Tainan, 709 Taiwan, Republic of China; 9 Graduate Institute of Medical Sciences, Chang Jung Christian University, Tainan, 711 Taiwan, Republic of China; 10 Department of Anesthesia, China Medical University, Taichung, 404 Taiwan, Republic of China; 11 Department of Biomedical Engineering, National Cheng Kung University, Tainan, 701 Taiwan, Republic of China

**Keywords:** Water-soluble fullerenol, Composition of exposed hydroxyl groups, Singlet oxygen quantum yield, Reactive oxygen species, Two-photon excitation

## Abstract

We successfully prepared water-soluble fullerenol [C_60_(OH)_46_] that exhibited a high singlet oxygen quantum yield and efficiently generated reactive oxygen species. Additionally, the water-soluble C_60_(OH)_46_ with a higher composition of exposed hydroxyl groups had superior two-photon stability and characteristics compared with that with a lower composition of such groups. Therefore, the prepared fullerenol can be an effective two-photon photosensitizer. The water-soluble C_60_(OH)_46_ had favorable two-photon properties. During two-photon photodynamic therapy, the water-soluble C_60_(OH)_46_ had substantial antimicrobial activity against *Escherichia coli* at an ultralow-energy level of 211.2 nJ pixel^−1^ with 800 scans and a photoexcited wavelength of 760 nm.

## Introduction

Various photosensitizer (PS) molecules have been synthesized over the past few decades [1]. However, clinical applications of existing PSs involve several problems. Most PS molecules are hydrophobic and can easily aggregate in aqueous media, thus reducing their quantum yield (QY) [2]. Moreover, aggregated PSs cannot simply be injected intravenously. Selective accumulation of PS molecules in deceased tissues is also required to prevent damage to healthy cells. Because of these problems, developing an effective PS carrier remains a major challenge to photodynamic therapy (PDT). Accordingly, interest in using nanoparticles as PS carriers is increasing.

Progress in nanobiotechnology has stimulated interest in the biomedical applications of a new class of nanostructures [3–11] that are composed exclusively of carbon atoms, namely fullerene C_60_, which are spheroidal molecules (0.72 nm in diameter) that are nontoxic and have unique physicochemical properties. The small size of lipophilic C_60_ molecules is responsible for their steric compatibility with biological molecules and promotes their integration into hydrophobic regions of membranes [12, 13]. Because of the extended *π*-conjugated system of its molecular orbitals, fullerene C_60_ absorbs ultraviolet–visible (UV–vis) light and can generate reactive oxygen species (ROS) with a nearly 100% singlet oxygen QY (*Φ*_Δ_). Furthermore, the physicochemical properties of fullerene C_60_ enable it to generate ROS and serve as a PS for PDT. Fullerenes may also induce prooxidant effects, and this might be dictated by the fullerene used, cell type investigated, and experimental setup [14–17]. C_60_ has extremely low solubility in polar solutions, which considerably limits its applications in medicine. However, because of the presence of double bonds, C_60_can be easily modified using chemical groups to increase its water solubility. Therefore, water-soluble C_60_ derivatives have increased opportunities for medical applications, including neuroprotection, drug and gene delivery, photosensitizing, and biosensing.

**Fig. 1:**
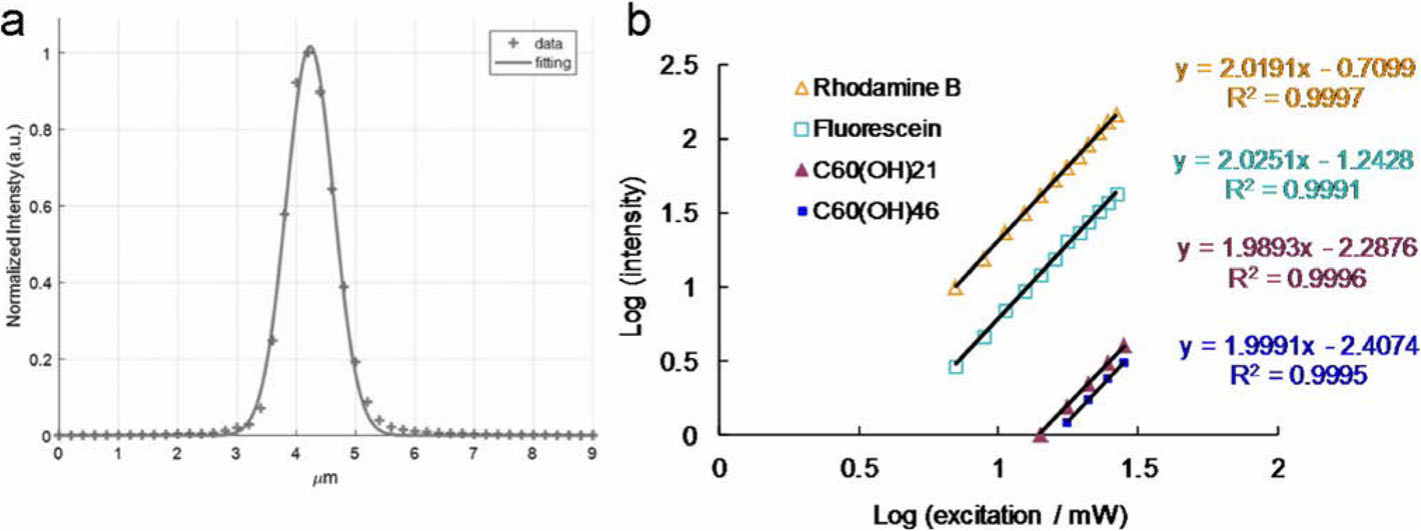
**a** According to the *z*-axis scan of a gold thin film used to measure the signal of second harmonic generation at different positions, the *z*-axis resolution of the laser system (full width at half maximum) is approximately 0.90159 μm (fitting using Gaussian function). **b** TPL intensity dependence on the excitation power (logarithm) of the materials and fluorophores; TPE exposure from 704.0 to 2816.0 nJ pixel^−1^ for Rhodamine B and fluorescein, from 1408.0 to 2816.0 nJ pixel^−1^ for water-soluble C_60_(OH)_21_ fullerenol, and from 1760.0 to 2816.0 nJ pixel^−1^ for water-soluble C_60_(OH)_46_ fullerenol. Excitation wavelength, 760 nm. Delivered dose: OD_600_ 0.05 of *E. coli* and 3 μg mL^−1^ materials. Data are presented as means ± SD (*n* = 6)

Multiphoton laser microscopy (also known as two-photon laser microscopy) entails the use of localized “nonlinear” excitation to excite fluorescence within only a thin raster-scanned plane. Two-photon laser microscopy has been used in various imaging studies [18]. It is typically coupled with near-infrared (NIR) laser excitation to capitalize on the inherent maximum tissue transmission for bioimaging; this is because NIR has the advantages of slight scattering, low-energy absorption, optimal irradiation penetration, and reduced photobleaching of specimens. The coupling of two-photon laser microscopy with NIR laser excitation has become the preferred technique for fluorescence microscopy in thick tissue and deeper biological specimens [19, 20] and has been extensively applied in other photoexcitation therapies [21, 22]. Moreover, because of its ultralow energy and short photoexcitation, two-photon laser microscopy is considered an alternative approach to performing PDT [23]. Although some PSs are toxic [24, 25], those with a high *Φ*_Δ_ are prioritized for conducting PDT. A high *Φ*_Δ_ value is particularly desirable when two-photon techniques are used to evaluate molecular activities in photoproperties and efficiently conduct nonlinear microscopic studies; such a value is desirable because the ratio of the energy absorbed to the input energy flux to a specimen is high, minimizing possible photodamage to the specimen [26]. However, the literature does not include studies that have considered the use of materials with two-photon properties for PDT. To fill this research gap, the present study applied water-soluble hydroxylated fullerenol with strong electron donation ability and a large *π*-conjugated system to increase charge transfer efficiency, thereby enhancing two-photon properties. Specifically, water-soluble hydroxylated C_60_(OH)_46_ was derived and applied as a two-photon PS for effective microbe removal using ultralow-energy femtosecond laser irradiation and only 800 scans under two-photon excitation (TPE; excitation wavelength, 760 nm). For the ultralow-energy femtosecond laser irradiation, the energy was 211.2 nJ pixel^−1^ and power was 2.112 mW (regarding the calculation of laser power after the objective, see the “Materials and Methods” section and Fig. 1a, where the *x*–*y* focal point and *z*-axis resolution of the laser system are approximately 0.37538 and 0.90159 μm, respectively); in addition, for the scanning process, the total effective exposure time was approximately 3.2621 s, scan rate was 4.0776 ms scan^−1^, and scan area was 200 × 200 μm^2^ (see the “Materials and Methods” section for details regarding the calculation). The water-soluble hydroxylated C_60_(OH)_46_ achieved a nearly 100% elimination of *Escherichia coli* (*E. coli*, a Gram-negative bacterial strain). Furthermore, the water-soluble C_60_(OH)_46_ with a higher composition of hydroxyl groups exhibited superior two-photon photoproperties compared with that with a lower composition of hydroxyl groups under TPE; therefore, the derived water-soluble C_60_(OH)_46_ can be considered to have considerable potential for use in simultaneous PDT to eliminate malignant microbes.

## Materials and Methods

### Preparation and Characterization of Water-Soluble Fullerenols, C_60_(OH)_21_ and C_60_(OH)_46_ [27]

Raw fullerene was obtained commercially (Sigma-Aldrich, St. Louis, MO, USA), and the C_60_(OH)_12_ precursor was produced, as previously described. First, 30% hydrogen peroxide solution (100 mL; Sigma-Aldrich, St. Louis, MO, USA) was added to the starting material, 0.5-1.0 g of C_60_(OH)_12_, and the mixture was vigorously stirred at 60 °C under air. After cooling, a mixture of solvents comprising 2-propanol, diethyl ether, and hexane (100–200 mL for each; Sigma-Aldrich, St. Louis, MO, USA) was added into the solution, which was subsequently centrifuged and decanted. The remaining solid was washed twice with 200 mL of diethyl ether through the centrifugation and decantation procedures. Finally, the end products of water-soluble C_60_(OH)_21_ and C_60_(OH)_46_ were obtained by drying the residue under vacuum at room temperature overnight, respectively. The weight of the end product was calibrated through thermal gravimetric analysis. The morphology of the end product was observed using a high-resolution transmission electron microscopy (HR-TEM, JEOL 3010, Akishima, Tokyo, Japan) at a resolution of approximately 1.11 ± 0.03 nm and 1.13 ± 0.04 nm for C_60_(OH)_21_ and C_60_(OH)_46_, respectively. The dynamic light scattering (DLS, Malvern Nano-ZS90, Worcestershire, West Midlands, UK) was also used to determine the size of materials. The exposed functional groups of the as-prepared materials were first examined through Fourier-transform infrared (FTIR) spectroscopy (RX1, PerkinElmer, Waltham, MA, USA). UV–vis spectroscopy of the materials was conducted using a spectrometer (U-4100, Hitachi, Chiyoda-ku, Tokyo, Japan). The surface chemistry of the fullerenol was examined through X-ray photoelectron spectroscopy (XPS, PHI 5000 spectrometer (VersaProbe, Chanhassen, MN, USA)). The molecular weight of fullerenol was determined using a field desorption (FD) mass spectrometer (AccuTOF, GCx-plus, JEOL, Akishima, Tokyo, Japan), and the number of hydroxyl groups was confirmed to be 21 and 46 based on the results, respectively.

### Bacterial Cultures [28]

*E. coli*, obtained from our own laboratory were grown in nutrient agar of LB (per liter: tryptone 10 g, yeast extract 5 g, sodium chloride 8 g, agar 15 g, and pH tuned to 7.5) (Sigma-Aldrich, St. Louis, MO, USA) and incubated at 37 °C.

### Biocompatibility Assay with Colony Forming Unit (CFU) Counting Method [28]

*E. coli* (OD_600_ ~ 0.05) was added with material (0–9 μg mL^−1^), and incubated for 3 h at 37 °C (Additional file 1: Fig. S1). After incubation, the mixture was centrifuged and the pellets of bacteria were diluted (OD_600_ ~ 0.05). A dilution factor of 10^−5^ to 10^−8^ was then conducted in the incubated bacteria and plated on the agar plates. The plates remain in an incubator (at 37 °C) overnight. The number of surviving bacteria was determined and expressed as a percentage (%) that corresponded to the unit of CFU mL^−1^ after incubation. Data are means ± SD (*n* = 6).

### *ψ*_Δ_ Measurement [29, 30]

According to the previous study, *ψ*_Δ_ can be obtained. *ψ*_Δ_ measurements were carried out in D_2_O at 355 nm, using *meso*-tetra(4-sulfonatophenyl)porphine dihydrochloride (TSPP; Sigma-Aldrich, St. Louis, MO, USA) as a reference (*ψ*_Δ_ = 0.64).

### Fluorescence QY Measurement [31, 32]

The relative photoluminescence (PL) QY of contrast agent is usually the ratio of the emitted photons to the absorbed photons and is given as follows:

1 $$ \mathrm{QY}={\mathrm{QY}}_{\mathrm{ref}}\ \left({\eta}^2/{\eta_{\mathrm{ref}}}^2\right)\left(I/A\right)\left({A}_{\mathrm{ref}}/{I}_{\mathrm{ref}}\right) $$

where QY_ref_ = 0.28 is the QY of Cy5.5 dissolved in dimethyl sulfoxide (DMSO; Sigma-Aldrich, St. Louis, MO, USA) as a reference, *η* is the refractive index of ddH_2_O = 1.33 (*η*_ref_ of DMSO = 1.48), *I* is the integrated fluorescence intensity and *A* is the absorbance at the excitation wavelength. One-photon excitation (OPE) or TPE yields the same QY.

### Femtosecond Laser Optical System for the Measurements of Two-Photon Absorption (TPA) and Two-Photon Luminescence (TPL) [23, 28, 33–38]

The home-made femtosecond titanium-sapphire (ti-sa) laser optical system (a repetition rate of 80 MHz; Tsunami, Spectra-Physics, Santa Clara, CA, USA) was used according to the previous studies.

#### TPA Measurement

With a galvanometer scanner speed of 2 m ms^−1^, the excitation spectrum was measured as 720–820 nm with an excitation power of 2.8 mW [this is the power before objective; the power after objective (or on sample) is 0.9856 mW or 98.56 nJ pixel^−1^]. Therefore, the relative TPA spectra as function of excitation wavelength for the fullerenols were measured.

#### Measurement of TPL Spectra

The material was exposed to TPE from the femtosecond laser at an excitation wavelength of 760 nm, a scanning area of 200 × 200 μm^2^, a frequency of 10 kHz, an exposure time of 1.638 s/(scan, pixel) = 100 μs, 128 × 128 pixels scan^−1^, and a pixel area of 1562.5 × 1562.5 nm^2^. The focal spot area was calculated as π*d*^2^/4, where *d* = 0.61 λ/numerical aperture (*NA*) is the full width at half maximum of the beam waist. For instance, at the *x*–*y* axis focal spot with 760 nm excitation and a × 40 oil-immersion objective with an *NA* of 1.3, *d* = 0.61 × 800 nm/1.3 = 375.38 nm = 0.37538 μm, and the *z*-axis resolution was measured to be 0.90159 μm. For 760 nm excitation, the exposure time per scan for an individual nanomaterial is expressed as (focal spot area/pixel area) × 100 = 4.0776 ms, and the total exposure time *t* = 4.0776 ms × number of scans. A × 40 oil-immersion objective (*NA* 1.3) was used to collect the signals, and the detection range of the spectrum photometer was 300–695 nm.

Additionally, the calculations of laser power (mW or nJ pixel^−1^) used on the sample were as follows. For the × 40 oil-immersion objective (*NA* 1.3), the transmission rate at 760 nm in wavelength is approximately 88% in this optical system, and the laser power went from the output to the objective with only 40% of the original output power due to the loss of power. As a result, the calculated energy after the objective (on sample) is *P*_output_ (mW)*40%*88% = 0.352 × *P*_output_ (mW). For instance, *P*_output_ = 2.8 mW, the calculated energy after the objective (on the sample) is 3.0 mW*40%*88% = 0.9856 mW. With 10 kHz of scan rate (each pulse stays 0.1 ms pixel^−1^), the calculated energy on the sample (J pixel^−1^) was around *P*_output_ (mW)*40%*88%*0.1 ms = 0.0352*P_output_ (J pixel^−1^). For instance, *P*_output_ = 2.8 mW, the energy (J pixel^−1^) on sample = 2.8 mW*40%*88%*0.1 ms = 0.09856 μJ pixel^−1^ = 98.56 nJ pixel^−1^. The power after the objective (on the sample) was used and marked throughput this manuscript.

### Measurement of TPE Absolute Cross Section [24, 36–48]

The absolute cross section of TPE was measured the luminescence signal *via* femtosecond laser optical system according to previous studies. The TPL of fluorescein and rhodamine B (Sigma-Aldrich, St. Louis, MO, USA) had to be verified. The results are shown in Fig. 1b and were obtained by measuring the dependence of the emission intensity with an excitation power range of 704 nJ pixel^−1^ (7.04 mW) to 2816 nJ pixel^−1^ (28.16 mW). Quadratic dependence with the exponents of 2.03 for fluorescein and 2.02 for rhodamine B was measured for increasing the excitation power to determine the luminescence from TPE. According to previous studies, the action cross sections of TPE for fluorescein and rhodamine B are 36.4 and 68.0 GM (1 GM = 10^−50^ cm^4^ s photon^−1^), respectively, for 760 nm excitation. We also referred to the free website http://www.drbio.cornell.edu/cross_sections.html, kindly provided by Prof. Chris Xu (Cornell University, NY, USA). The TPE action cross sections for fluorescein and rhodamine B were calculated to be 36.5 and 66.1 GM, respectively (Table 1), which indicated an error of less than 5% compared with those from Prof. Xu’s laboratory. In this study, rhodamine B was chosen as the standard reference for determining the cross section, and the calculated absolute cross sections of TPE for the water-soluble C_60_(OH)_21_ and C_60_(OH)_46_ fullerenols were approximately 1230.51 GM and 1037.21 GM, respectively. The measured parameters for calculating the TPE absolute cross sections of samples are shown in Table 3. No batch-to-batch variation was observed for the materials in two-photon properties and two-photon photodynamic ability.

**Table 1:** Two-photon action cross sections of fluorescein (in 0.1 M NaOH, pH 11) and rhodamine B (in methanol). Excitation wavelength, 760 nm

	Fluorescein (in ddH_2_O, pH = 11)	Rhodamine B (in methanol)
Excitation wavelength at 760 nm action cross section, ησ_2_ (GM, 10^−50^cm^4^s/photon)	36.5	66.1

### Femtosecond Laser Optical System (for Fluorescence Lifetime Imaging Microscopy, FLIM) [39, 45]

The home-made femtosecond ti-sa laser optical system (repetition rate of 80 MHz; Tsunami, Spectra-Physics, Santa Clara, CA, USA) was used according to the previous studies. The lifetime data and parameter are generated using the triple-exponential equation fitting while monitoring the emission under TPE (Ex, 760 nm).

### Calculation of Radiative and Nonradiative Decay Rates [46]

PL QY and lifetime are both major parameters when investigating the emission characteristics of fluorescent dyes in diverse environments. The QY (*Q*) can be expressed as follows:

2 $$ Q=\frac{\varGamma }{\varGamma +k} $$

where *Γ* is the radiative decay rate, and *k* is the nonradiative decay rate. Fluorescence lifetime is usually defined as the average time required for an electron in the excited state to decay to the ground state. The TPL lifetime *τ* can also be relative to the decay rates and is described as follows:

3 $$ \tau =\frac{1}{\varGamma +k} $$

Following Eqs. (2) and (3), the radiative and nonradiative decay rates can be calculated.

Upon the absorption of a photon, one of the weakly bound electrons of the fluorescent molecule—a fluorophore—is promoted to a higher energy level. The fluorophore is then in an excited state, A*. This state is metastable; therefore, the fluorophore will return to its stable ground state, *A*. It can do so either radiatively by emitting a fluorescence photon *hν*

$$ A\ast ->A+ h\nu $$

or nonradiatively by dissipating the excited state energy as heat:

$$ A\ast ->A+\mathrm{heat} $$

The depopulation of the excited state depends on the de-excitation pathways available. Fluorescence is radiative deactivation of the lowest vibrational energy level of the first electronically excited singlet state, *S*_*1*_, back to the electronic ground state, *S*_*0*_. The singlet states are the energy levels that can be populated by the weakly bound electron without a spin flip. The absorption and emission processes are illustrated by an energy level diagram named after Aleksander Jablonski.

The fluorescence lifetime, *τ*, is the average time a fluorophore remains in the electronically excited state *S*_*1*_ after excitation. *τ* is defined as the inverse of the sum of the rate parameters for all excited state depopulation processes: Eq. (3), where the nonradiative rate constant *k* is the sum of the rate constant for internal conversion *k*_ic_ and the rate constant for intersystem crossing to the triplet state *k*_isc_ such that *k* = *k*_ic_ + *k*_isc_. Fluorescence emission always occurs from the lowest vibrational level of *S*_1_, a rule known as the Kasha’s rule, indicating that the fluorophore has no memory of its excitation pathway; for example, OPE and TPE yield the same fluorescence spectrum, QY, and lifetime.

### Determination for Bacteria Viability Rates After Laser Exposure [28]

#### CFU Counting Method

Bacteria (OD_600_ ~ 0.05) was added with material (3 or 6 μg mL^−1^) by incubating for 3 h at 37 °C in darkness. After incubation, the mixture was centrifuged and the pellets of bacteria were diluted (OD_600_ ~ 0.05) and exposed to a TPE power of 211.2 nJ pixel^−1^ with 800 scans (approximately 3.2621 s of total effective exposure time; Ex, 760 nm). Then, a dilution factor of 10^−5^ to 10^−8^ was then conducted in the incubated bacteria and plated on the agar plates. The plates remain in an incubator (at 37 °C) overnight. The number of surviving bacteria was determined and expressed as a percentage (%) that corresponded to the unit of CFU mL^−1^ after incubation. Data are means ± SD (*n* = 6).

#### LIVE/DEAD Kit

Bacteria (OD_600_ ~ 0.05) was added with material (3 or 6 μg mL^−1^) by incubating for 3 h at 37 °C in darkness. After incubation, the mixture was centrifuged and the pellets of bacteria were diluted (OD_600_ ~ 0.05) and exposed to a TPE power of 211.2 nJ pixel^−1^ with 800 scans (approximately 3.2621 s of total effective exposure time; Ex, 760 nm). Then, the pellets were stained using a LIVE (SYTO 9, as displayed with green fluorescence)/DEAD (*propidium iodide,* PI, as displayed with red fluorescence) kit (Thermo Fisher Scientific, Waltham, MA, USA) according to the instruction. The viability of bacteria was quantified for antimicrobial tests, which showed nearly all nanomaterial-treated bacteria to be dead after treatment. Similar viability was quantified through the CFU counting method to determine the efficient antibacterial effects of materials in PDT. Data are presented as mean ± SD (*n* = 6).

### ROS Detection [23, 29, 34, 35, 49–55]

#### Singlet Oxygen (^1^O_2_)

(a) Material (3 or 6 μg mL^−1^) was treated with bacteria (OD_600_ ~ 0.05), after which it was subjected to 3 h of incubation at 37 °C in darkness. Subsequently, the mixture was exposed to TPE photoexcitation (211.2 nJ pixel^−1^, 800 scans; Ex, 760 nm) and finally mixed with Singlet Oxygen Sensor Green (SOSG) reagent (1 μM; Thermo Fisher Scientific, Waltham, MA, USA) (Ex/Em: 488/525 nm). A fluorescence spectrometer was employed for measurements. For ROS neutralization, the mixture was mixed with 30 ppm of antioxidant *α*-tocopherol/methyl linoleate (Sigma-Aldrich, St. Louis, MO, USA) in darkness and exposed to TPE photoexcitation with the same treatment. (b) Material (3 or 6 μg mL^−1^) was treated with bacteria (OD_600_ ~ 0.05), after which it was subjected to 3 h of incubation at 37 °C in darkness. Subsequently, the mixture was exposed to TPE photoexcitation (211.2 nJ pixel^−1^, 800 scans; Ex, 760 nm) and finally mixed with 10 μM of trans-1-(2′-methoxyvinyl)pyrene (*t*-MVP, Thermo Fisher Scientific, Waltham, MA, USA)/0.10 M SDS (Sigma-Aldrich, St. Louis, MO, USA) (Ex/Em: 352/465 nm). For ROS neutralization, the mixture was mixed with 30 ppm of antioxidant *α*-tocopherol/methyl linoleate (Sigma-Aldrich, St. Louis, MO, USA) in darkness. Reaction of *t*-MVP with ^1^O_2_ yields a dioxetane intermediate that fluoresces while it decomposes into 1-pyrenecarboxaldehyde. Furthermore, this highly selective fluorescent probe does not react with other activated oxygen species such as hydroxyl radicals, superoxide, or hydrogen peroxide. A fluorescence spectrometer was employed for measurements. ROS neutralization was conducted with the same as previously described treatment.

#### Superoxide Radical Anion (O_2_^.−^)

(a) Material (3 or 6 μg mL^−1^) was treated with bacteria (OD_600_ ~ 0.05), after which it was subjected to 3 h of incubation at 37 °C in darkness. Subsequently, the mixture was exposed to TPE photoexcitation (211.2 nJ pixel^−1^, 800 scans; Ex, 760 nm) and finally mixed with 2, 3-bis (2-methoxy-4-nitro-5-sulfophenyl)-2H-tetrazolium-5-carboxanilide (XTT, 0.45 mM; Sigma-Aldrich, St. Louis, MO, USA). The purpose of this material was that it interacted with O_2_^**.**−^ and produced XTT-formazan, resulting in strong absorption (470 nm in wavelength). UV–vis spectrometer was employed to monitor this absorption. For ROS neutralization, the mixture was mixed with 30 ppm of antioxidant *α*-tocopherol/methyl linoleate (Sigma-Aldrich, St. Louis, MO, USA) in darkness and exposed to TPE photoexcitation with the same treatment. (b) Material (3 or 6 μg mL^−1^) was treated with bacteria (OD_600_ ~ 0.05), after which it was subjected to 3 h of incubation at 37 °C in darkness. Subsequently, the mixture was exposed to TPE photoexcitation (211.2 nJ pixel^−1^, 800 scans; Ex, 760 nm) and finally mixed with 50 mM bicarbonate buffer (pH 8.60) and glutathione (*γ*-l-glutamyl-l-cysteinyl-glycine, GSH, Sigma-Aldrich, St. Louis, MO, USA)/0.80 mM bicarbonate buffer (the Ellman’s assay for O_2_^**.**−^ detection). Subsequently, the following experiments were conducted according to the procedure in a previous study. Loss of GSH (%) was calculated as the difference in absorbance between the sample and negative control divided by the absorbance of the negative control. The signal of the generated O_2_^**.**−^ was obtained as described in the previous calculation. Data are means ± SD (*n* = 6).

### Uptake Assay [35]

*E. coli* (OD_600_ ~ 0.05) were incubated with 3 μg mL^−1^ material. The absorbance of a quantity of 3 μg mL^−1^ material was recorded by UV–vis spectroscopy (Abs, approximately 203 nm). The materials were mixed with *E. coli* (OD_600_ ~ 0.05) at 37 °C from the 1st hour to the 10th hour, respectively, and centrifuged (1200 rpm) to remove excess materials and keep the supernatant and measure its absorbance. The difference in absorbance between the collected supernatant and the original materials was estimated, resulting in the percentage of uptake at each time point. Data are means ± SD (*n* = 6).

### Statistical Analysis [56]

The statistical significance was by the analysis of variance. The *p* value was considered statistically significant for all the treatments.

## Results and Discussion

### Characterization of Water-Soluble Fullerenol

**Fig. 2:**
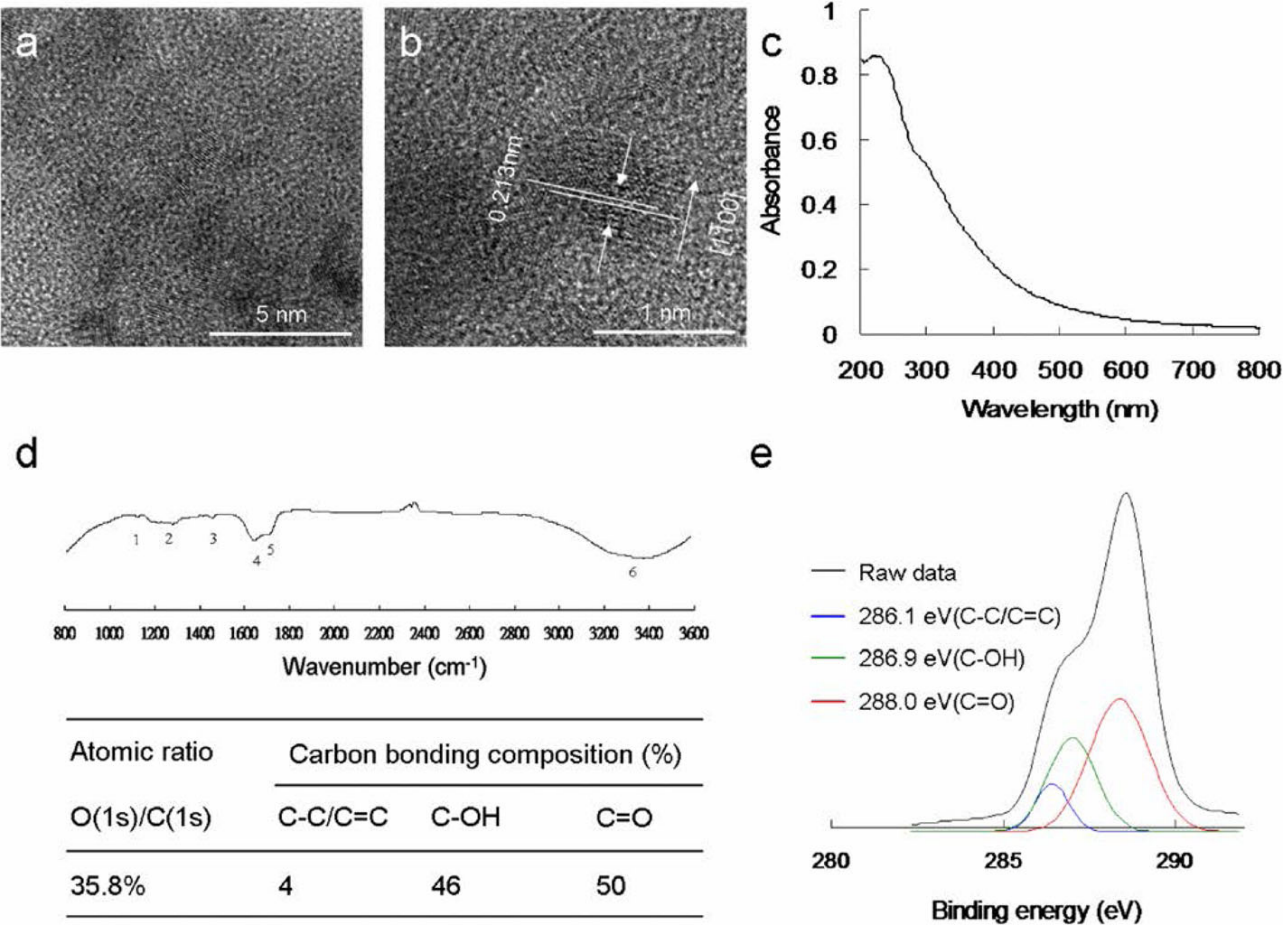
Functional characterization of the synthesized water-soluble C_60_(OH)_46_ fullerenol. **a** Low-magnified TEM image and **b** HR-TEM image of a water-soluble fullerenol illustrating the materials {1$\overline{1}$00} lattice planes and the mean size of 1.11 ± 0.03 nm with a *d*-spacing of 0.213 nm. **c** UV–vis and **d** FTIR spectra of nanomaterial. **e** Deconvoluted C(1s) XPS spectra and fitted peaks obtained using Gaussian function: nonoxygenated ring (C–C/C=C), C–O bond, and C=O bond, respectively. The atomic ratio and bonding composition of fullerenol are shown as summarized in the table. The O(1s)/C(1s) atomic ratio is 35.8%

Water-soluble C_60_(OH)_46_ (fullerenol), which was determined to be circular and monodispersed, was synthesized in accordance with a previous study [27]. The mean lateral size of the fullerenol was approximately 1.13 ± 0.04 nm, as determined using low-magnification (Fig. 2a) and HR-TEM images (Fig. 2b). Furthermore, the fullerenol was noted to exhibit favorable crystallinity along with a good lattice spacing, which corresponded to the *d*-spacing of the fullerenol {1$\overline{1}$00} lattice fringes. However, these particles could form aggregates through hydrogen bonding in an aqueous solution with a pH of 7.0. The average size of the formed aggregates was approximately 130 nm, as revealed by a DLS analysis. Moreover, the aggregates remained highly stable for 3 months in different physiological environments, such as a pH 7.0 aqueous solution, 1× phosphate-buffered saline, and culture medium (Additional file 1: Table S1). In the UV–vis absorption spectrum of the fullerenol, absorbance peaks were observed at approximately 216 and 309 nm, and these peaks were attributed to the *π*–π* transition of aromatic C=C bonds and the *n*–π* transitions of the C=O shoulder, respectively. The *π*-electron transition in the fullerenol contained oxygen (Fig. 2c), as is typically observed for aqueous dispersions, thereby confirming the presence of the fullerenol. Additional characterizations were performed using FTIR, XPS, and mass spectrometry to confirm the properties of the prepared materials. FTIR was used to analyze the exposed functional groups of the prepared materials. The analysis results revealed the following characteristic material bands: a C–O stretching band at approximately 1109 cm^−1^ (band 1), phenolic C–OH stretching band at approximately 1271 cm^−1^ (band 2), tertiary alcoholic C=O stretching band at approximately 1422 cm^−1^ (band 3), C=C stretching band at approximately 1674 cm^−1^ (band 4), C=O stretching band at approximately 1721 cm^−1^ (band 5), and C–H intermolecular hydrogen-bonded and carboxylate O–H stretching band at approximately 3318 cm^−1^ (band 6). Furthermore, a band of CO_2_ interference was observed. These bands revealed exposed hydroxyl and carbonyl groups as well as aromatic C=C bonds (Fig. 2d). XPS was performed to examine the surface chemistry of the fullerenol, which predominately contains carbon atoms in general. The deconvoluted C(1s) spectra of the fullerenol revealed a nonoxygenated ring (C–C/C=C, 286.1 eV), C–O bond (286.9 eV), and C=O bond (288.0 eV). In addition, the O(1s)/C(1s) ratio was approximately 35.8% (Fig. 2e). The molecular weight of the fullerenol was also determined using FD mass spectrometry (Additional file 1: Fig. S2); the number of hydroxyl groups (C–OH) was confirmed to be 46, which was consistent with the atomic ratios and bonding compositions of the fullerenol summarized in Fig. 2. These characterization results confirm the successful synthesis of fullerenol.

### ROS Generation of Water-Soluble Fullerenol Under TPE

**Fig. 3:**
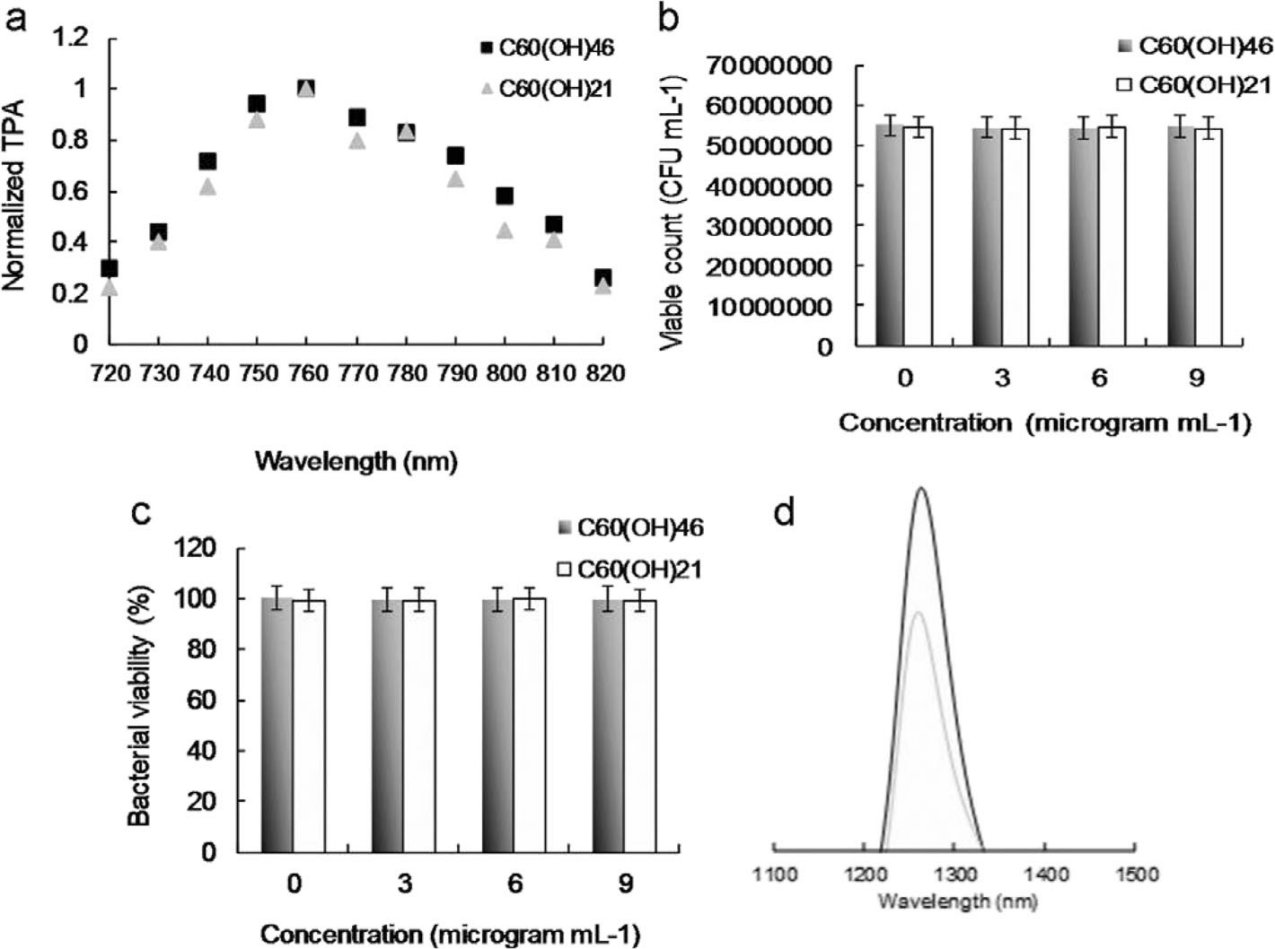
**a** Relative TPA spectra of the material. TPE as a function of the wavelength (720–820 nm) at 98.56 nJ pixel^−1^ that was used to monitor the signals. Delivered dose, 3 μg mL^−1^ water-soluble C_60_(OH)_46_ or C_60_(OH)_21_ fullerenol. The number of surviving **b** material-treated bacteria was determined by CFU counting assay and is expressed as the percentage (%) for **c** bacteria that corresponds to the unit of CFU mL^−1^. Delivered dose, OD600 ~ 0.05 of *E. coli* and 0–9 μg mL^−1^ water-soluble fullerenol. **d** Measurement of phosphorescence spectra at 1270 nm for material. Delivered dose, 3 μg mL^−1^ water-soluble fullerenol. Data are means ± SD (*n* = 6)

A PS absorbs and transfers light energy to other nonabsorbing molecules to generate ROS, which kill targeted cells, damage tumor vasculature, and activate an antitumor immune response. PSs have a particular arrangement of electrons in their molecular orbitals. Similar to nearly all molecules, at ground (singlet) state, PSs have couples of electrons with opposite spins in low-energy molecular orbitals. The absorption of light at an appropriate wavelength lifts an electron to a high-energy orbital without changing its spin. This is a short-lived (nanoseconds) excited singlet (S_1_) state, and the PS can lose its energy and return to the ground state by emitting light (fluorescence) or heat. Alternatively, intersystem crossing, wherein the spin of the excited electron is inverted, can occur in the S_1_ state. This electron spin inversion is responsible for the relatively long life (lasting microseconds) of the excited triplet (T_1_) state. Radiative triplet-to-singlet transitions are inhibited because they require a change in electron spin, which is a slow process. From the T_1_ state, the PS can return to the ground state by emitting light (phosphorescence) or transferring energy to another molecule. It can also lose energy through internal conversion or radiationless transitions when colliding with other molecules. The longer the life of the PS in the T_1_ state is, the higher are its chances of colliding with another molecule, resulting in ROS production [57–59]. The photosensitization of water-soluble fullerenols results in their transition to a long-lived T_1_ state and subsequent energy or electron transfer to molecular oxygen, yielding ROS such as ^1^O_2_ and O_2_^**.**−^, which have major roles in PDT. Therefore, ^1^O_2_ and O_2_^**.**−^ produced by water-soluble C_60_(OH)_46_ must be detected directly using laser irradiation. To detect ^1^O_2_ and O_2_^**.**−^ formation during PDT, in this study, PDT was initiated by combining excited the triplet water-soluble C_60_(OH)_46_, oxygen, and light configured to a suitable wavelength and energy as well as by introducing SOSG, *t*-MVP, XTT, and GSH reagents [33, 34, 49–51]. To exploit the potential bactericidal capability of the materials, a wavelength of approximately 760 nm was determined to be the most efficient for deriving the relative maximum TPA ratio of the water-soluble C_60_(OH)_46_ under TPE (Fig. 3a); this is attributable to the interband transitions involved [52]. This wavelength was used in subsequent experiments in this study. The water-soluble C_60_(OH)_46_ was photoexcited through TPE at a power of 211.2 nJ pixel^−1^ with 800 scans (Ex, 760 nm; total effective exposure time, ~ 3.2621 s) and delivered dose of 3 or 6 μg mL^−1^ (Additional file 1: Table S2). Furthermore, to confirm the involvement of ROS in the PDT effects of the water-soluble C_60_(OH)_46_, *α*-tocopherol was used for ROS neutralization [49, 53]. The quantity of generated ROS was reduced after the addition of *α*-tocopherol, but the observed bacterial viability increased as expected. Additionally, the quantity of generated ROS depended on the delivered dose. To prevent ^1^O_2_ and O_2_^**.**−^ production possibly engendered by inadvertent exposure of water-soluble C_60_(OH)_46_ to white light—which could have compromised the experiments in this study [60]—subsequent PDT experiments were conducted in the dark. This study focused on the quantities of generated ^1^O_2_ and O_2_^**.**−^. The water-soluble C_60_(OH)_46_ exhibited considerable antibacterial effects, demonstrating its potential for application in PDT. Notably, after the same experiment, the water-soluble C_60_(OH)_21_ (Additional file 1: Figs. S3, S4; Fig. 3a) was less effective in forming ^1^O_2_ and O_2_^**.**−^ when compared with the water-soluble C_60_(OH)_46_ (Additional file 1: Table S2). The water-soluble C_60_(OH)_46_ generated more ^1^O_2_ and O_2_^**.**−^ than did the water-soluble C_60_(OH)_21_; additionally, the water-soluble C_60_(OH)_46_ and water-soluble C_60_(OH)_21_ had *Φ*_Δ_ values of approximately 0.93 and 0.85, respectively (for reference, *Φ*_Δ_ = 0.64 is the QY of TSPP dissolved in D_2_O [29, 30]).

### Antimicrobial Ability Determination Using TPE

**Fig. 4:**
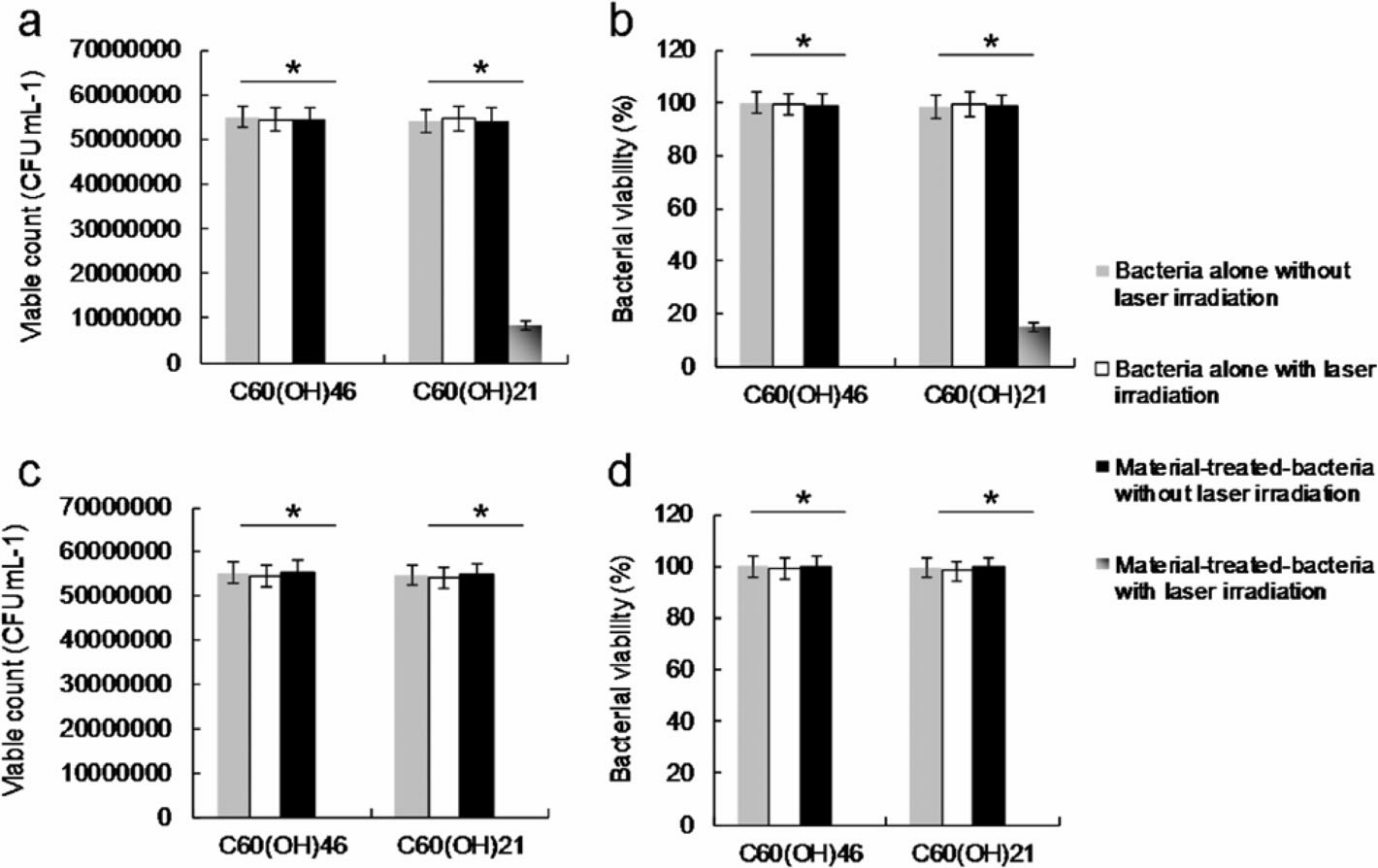
Viability (%) was quantified according to the determined viable count of material-treated bacteria through a CFU assay conducted using short excitation with a TPE power of 211.2 nJ pixel^−1^ with 800 scans (approximately 3.2621 s of total effective exposure time; Ex, 760 nm) to deliver a dose of **a**, **b** 3 or **c**, **d** 6 μg mL^−1^. Delivered dose, OD600 0.05 of *E. coli*. Data are presented as means ± SD (*n* = 6). For C_60_(OH)_46_- and C_60_(OH)_21_-treated *E. coli* with photoexcitation, **a***p* < 0.001 and *p* = 0.662, **b***p* < 0.001 and *p* = 0.658, **c***p* < 0.001 and *p* < 0.001, and **d***p* < 0.001 and *p* < 0.001. *p value obtained by Student’s *t* test

Before the execution of antimicrobial experiments, the toxicity of water-soluble fullerenols must be examined to exclude factors that could contribute to bacterial elimination and confound experimental results. In addition, to prevent possible ROS production engendered by the inadvertent exposure of experimental materials to white light, which could confound experimental results [35], PDT experiments must be conducted in the dark. This study applied Gram-negative *E. coli* as the experimental template. A CFU counting assay was conducted to determine the number of surviving bacteria (expressed herein as a percentage, corresponding to CFU mL^−1^). The bacteria were treated with two types of the prepared water-soluble fullerenols (dose range, 0 to 9 μg mL^−1^) and incubated in the dark for 3 h at 37 °C to determine absorbance at 600 nm (OD_600_ ~ 0.05; Additional file 1: Fig. S1). The growth levels of the bacteria treated with the water-soluble fullerenols were first monitored by measuring absorbance at 600 nm. The initial absorbance was 0.05 OD_600_, and the absorbance associated with both materials reached approximately 0.37 over time. Accordingly, neither material inhibited bacterial proferation. Moreover, the materials engendered a nearly 0 log_10_ reduction in the number of surviving bacteria (Fig. 3b), corresponding to a viability of approximately 100% (Fig. 3c). Accordingly, the materials were determined to exhibit excellent biocompatibility with the bacteria. Consequently, the materials subjected to 3 h of incubation in the dark at 37 °C were used to conduct experiments. Although the water-soluble fullerenol could generate ROS, interactions between materials and reagents (i.e., SOSG, *t*-MVP, XTT, and GSH) may result in false-positive ROS signals, thereby confounding PDT results [52]. Therefore, to exclude this possibility, bacteria were introduced and treated with materials in the present study. The amount of ROS generated from the photoexcited material-treated *E. coli* was observed. Table 2 presents the observed amount of ROS, revealing a similar trend to that in Tables S2–S3 (Additional file 1: materials alone and material-treated-Gram-positive *Bacillus subtilis* (*B. subtilis*)); these results were consistent with the ^1^O_2_ phosphorescence signal emitted from the materials at 1270 nm (Fig. 3d). PDT against *E. coli* was performed using irradiation with a low dose of energy (211.2 nJ pixel^−1^ with 800 scans, total effective exposure time ~ 3.2621 s; Ex, 760 nm). The effects PDT on the viability of *E. coli* treated with two-photon photoexcited materials were then determined (Fig. 4). No bactericidal effects were observed on bacteria alone (with or without laser exposure) or on the panel of material-treated bacteria without laser treatment (Fig. 4a). After TPE, bacterial viability was relatively low; specifically, the viability observed for the panel that was treated with the water-soluble C_60_(OH)_21_ was nearly 15%, corresponding to an approximately 0.823 log_10_ reduction (Fig. 4b). By contrast, the bacterial viability observed for the panel treated with the water-soluble C_60_(OH)_46_ was approximately 0 (100% elimination efficiency, corresponding to a ~ 7.736 log_10_ reduction). When the dose was increased, complete bactericidal effects were observed for both materials (Fig. 4c, d). However, antimicrobial effects did not differ by bacteria type (Gram-negative *E. coli* or Gram-positive *B. subtilis*) after photoexcitation (Additional file 1: Fig. S5). In addition, regarding the fullerenols that eliminated bacteria, a higher composition of hydroxyl groups increased bactericidal capability when compared with a lower composition under identical treatment conditions.

**Table 2:** The amount of ROS generated [23, 29, 33–35, 49–55] from by a TPE (211.2 nJ pixel^−1^, 800 scans; Ex, 760 nm) to water-soluble fullerenol-treated *E coli.* (3 or 6 μg mL^−1^) was conducted in the dark and monitored. Data are means ± SD (*n* = 6)

	^1^O_2_ (by SOSG)^c^						
	Negative control^ac^	ROS neutralization^abc^	Positive control^cd^	C_60_(OH)_46_	ROS neutralization^bc^	C_60_(OH)_21_	ROS neutralization^bc^
3 μg mL^−1^	33 ± 12	234 ± 12	2835 ± 135	2581 ± 115	233 ± 11	2235 ± 104	234 ± 10
6 μg mL^−1^	232 ± 11	233 ± 10	2841 ± 148	2626 ± 118	232 ± 13	2276 ± 109	232 ± 12
	^1^O_2_ (by *t*-MVP)^e^						
	Negative control^ae^	ROS neutralization^abe^	Positive control^de^	C_60_(OH)_46_	ROS neutralization^be^	C_60_(OH)_21_	ROS neutralization^be^
3 μg mL^−1^	340 ± 21	341 ± 22	9301 ± 228	8865 ± 199	340 ± 20	8547 ± 165	341 ± 21
6 μg mL^−1^	341 ± 20	339 ± 23	9316 ± 231	8942 ± 208	339 ± 18	8619 ± 172	340 ± 19
	O_2_˙^−^ (by XTT)^f^						
	Negative control^af^	ROS neutralization^abf^	Positive control^df^	C_60_(OH)_46_	ROS neutralization^bf^	C_60_(OH)_21_	ROS neutralization^bf^
3 μg mL^−1^	0	0	1.95 ± 0.15	1.86 ± 0.10	0.03 ± 0.01	1.77 ± 0.08	0.02 ± 0.01
6 μg mL^−1^	0	0	1.99 ± 0.17	1.90 ± 0.11	0.03 ± 0.02	1.81 ± 0.08	0.02 ± 0.02
	O_2_˙^−^ (by GSH)^g^						
	Negative control^ag^	ROS neutralization^abg^	Positive control^dg^	C_60_(OH)_46_	ROS neutralization^bg^	C_60_(OH)_21_	ROS neutralization^bg^
3 μg mL^−1^	0	0	98.9 ± 4.3%	84.6 ± 3.4%	0.3 ± 0.1%	76.7 ± 3.2%	0.2 ± 0.1%
6 μg mL^−1^	0	0	99.4 ± 4.7%	88.2 ± 3.7%	0.2 ± 0.1%	80.4 ± 3.6%	0.1 ± 0.1%

^a^Negative control: only treat reagent and laser radiation without material (0 μg mL^−1^)

^b^ROS neutralization: with the treatments of nanomaterial, the laser irradiation and 30 ppm of antioxidant α-Tocopherol/methyl linoleate

^c^SOSG reagent (Ex/Em, 488/525 nm) has a specific reactivity to generate fluorescence recorded by a PL spectrometer

^d^Positive control: the treatment of 50 μM *tert*-butyl hydroperoxide and laser irradiation

^e^*t*-MVP (Ex/Em, 352/465 nm) can react with ^1^O_2_, forming a dioxetane intermediate that generates fluorescence upon decomposition to 1-pyrenecarboxaldehyde, and monitored by a PL spectrometer

^f^XTT would interact with O_2_^**.**−^ and produce the XTT-formazan generating strong absorption (470 nm in wavelength)

^g^GSH containing a thiol-tripeptide can prevent damages to cellular or bacterial components caused by stress of oxidation. Thiol group from GSH can be oxidized to disulfide bond converting GSH to glutathione disulfide. GSH oxidation was used to determine the generated O_2_^**.**−^. Loss of GSH (%) = (absorbance difference between sample and negative control/absorbance of negative control) × 100%

### Observation of Water-Soluble Fullerenol-Treated *E. coli* Using TEM and Investigation of Two-Photon Properties

**Fig. 5:**
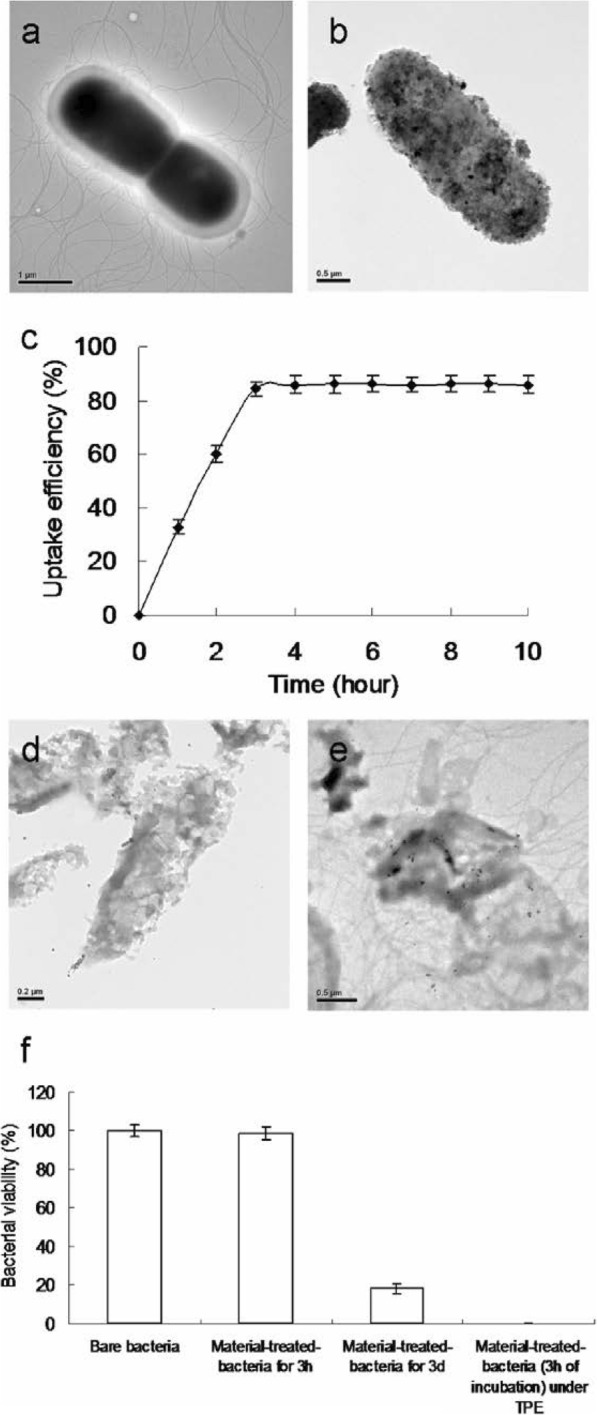
TEM images. **a** Showing bare bacteria without any treatment. Bacteria treated with material for **b** 3 h and **d** 3 days of incubation. **e** The photoexcited material-treated bacteria (3 h of incubation) with a TPE power of 211.2 nJ pixel^−1^ with 800 scans (approximately 3.2621 s of total effective exposure time; Ex, 760 nm). **c** Uptake assay of bacteria and material at 37 °C. **f** Viability (%) was quantified following the determined viable count of material-treated bacteria via CFU assay by short excitation with the same treatment. Delivered dose OD600 ~ 0.05 of *E. coli* and 3 μg mL^−1^ water-soluble fullerenol C_60_(OH)_46_. Data are means ± SD (*n* = 6)

**Fig. 6:**
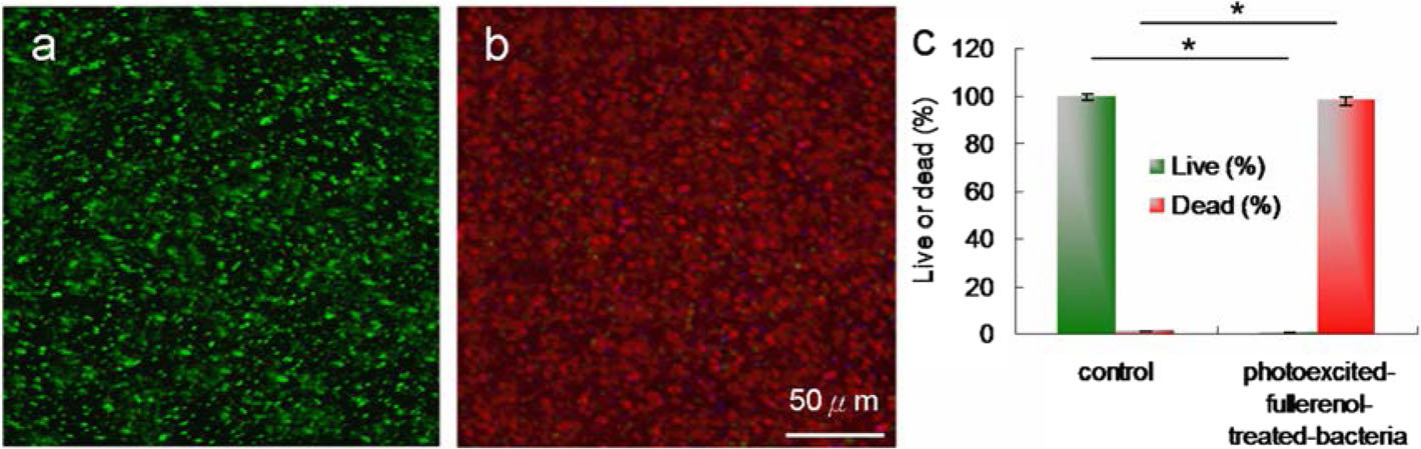
Images obtained after laser photoexcitation exposure (211.2 nJ pixel^−1^) with 800 scans (approximately 3.2621 s of total effective exposure time; Ex, 760 nm) of **a**, **b** material-treated bacteria. The Live/Dead kit was used to stain bacteria before images were obtained. Scale bar, 50 μm. **c** Viability (%) determination results. Delivered dose, OD600 ~ 0.05 of *E. coli* and 3 μg mL^−1^ water-soluble fullerenol C_60_(OH)_46_. For the percentages alive and dead, *p* < 0.001. *p value obtained using Student’s *t* test. Data are presented as mean ± SD (*n* = 6)

**Fig. 7:**
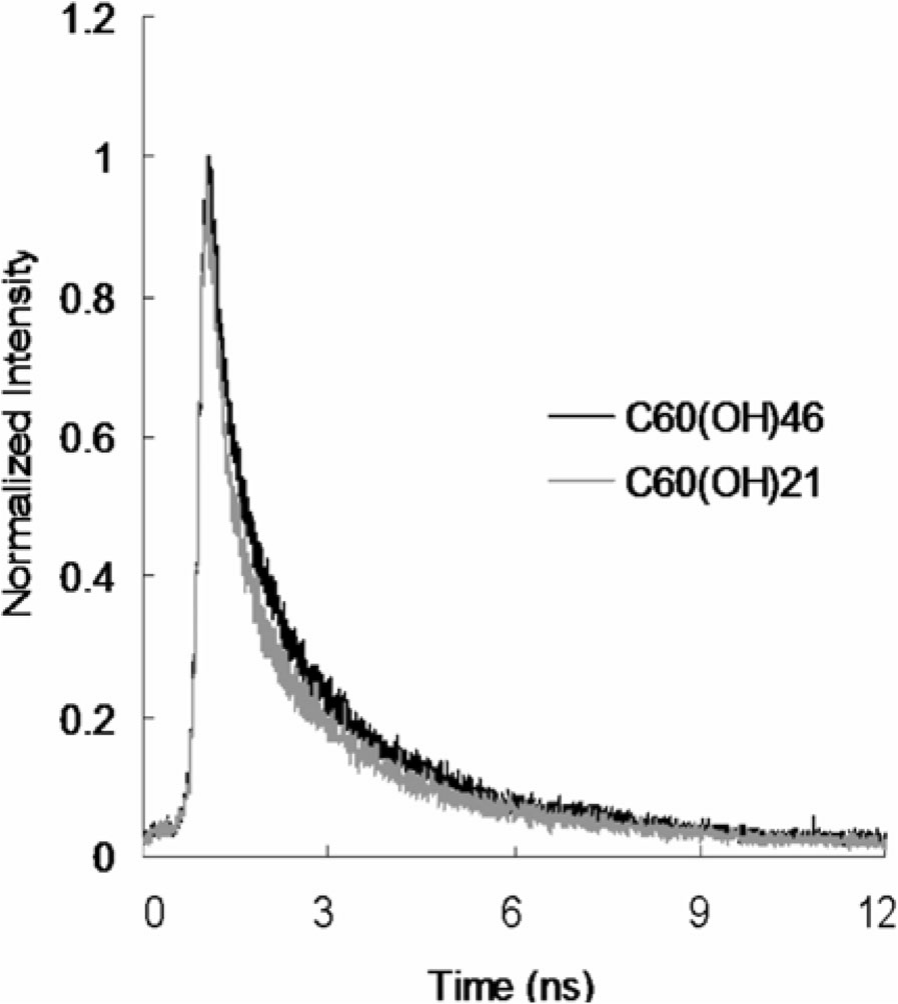
Time-resolved room-temperature PL decay profiles of material (98.56 nJ pixel^−1^). Excitation wavelength, 760 nm. Delivered dose, OD_600_~ 0.05 of *E. coli* and 3 μg mL^−1^ materials. Data are presented as means ± SD (*n* = 6)

To observe the disruption of material-treated bacteria after photoexcitation, the water-soluble C_60_(OH)_46_ with high PDT efficiency was selected, and bacteria were imaged using TEM. Bare *E. coli* (Fig. 5a) were incubated with the water-soluble fullerenol for 3 h, resulting in the substantial adsorption of materials on the bacterial surfaces. Nevertheless, no unusual morphologies were observed, indicating normal live bacterial morphology (Fig. 5b). Uptake assay results revealed the adsorption of materials onto the bacterial surface, with the corresponding burst rate being approximately 85% within the first 3 h of incubation (Fig. 5c); the rate reached saturation from the 3rd to the 10th hour. Therefore, the materials were adsorbed and formed an external barrier on the bacterial surface. However, the *E. coli* exhibited a distorted appearance and severe morphological changes over 3 days of incubation (Fig. 5d), resulting in a 0.940 log_10_ reduction that corresponded to a nearly 18% viability (Fig.5f; Additional file 1: Fig. S6). Material absorption and coating on the bacterial surface suppressed the absorption of nutrients essential for microbial growth and engendered changes in membrane (wall) permeability, thereby inducing internal osmotic imbalances and inhibiting microbial growth. In other words, the water-soluble fullerenol had antibacterial (bacteriostatic or bactericidal) effects after 3 days of incubation. Furthermore, the photoexcited material-treated bacteria, particularly *E. coli*, exhibited unique morphologies with severe damage after 3 h of incubation (Fig. 5e, f; Additional file 1: Fig. S6). No heat-generated bubbles formed on the bacterial surface incurred damage, indicating that the water-soluble fullerenol did not have photothermal-mediated heat properties after photoexcitation (Additional file 1: Fig. S7). The viability of *E. coli* was also determined through fluorescence and quantification (Fig. 6). The green fluorescence indicative of living bacteria in Fig. 6a reveals that the bacteria exposed to laser treatment alone were largely undamaged, which is consistent with the results presented in Fig. 5a. Dead bacteria were detectable after treatment with the materials and laser exposure (red fluorescence in Fig. 6b), a finding that is also consistent with that in Fig. 5e. Bacterial viability was quantified for further antimicrobial testing. Nearly complete elimination of the material-treated bacteria (Fig. 6c) was observed. Viability was also quantified using a CFU assay (Figs. 4a, b and 5f, and Additional file 1: Fig. S6) to demonstrate the antibacterial efficiency of the water-soluble C_60_(OH)_46_ in PDT. According to the results in Figs. 4, 5, and 6; Table 2; and Table S2 (Additional file 1), *E. coli* treated with the water-soluble C_60_(OH)_46_ was susceptible to photoexcitation, leading to a higher death rate, increased ROS generation, and more severe morphological collapse compared with *E. coli* treated with the water-soluble C_60_(OH)_21_. In general, the absolute cross section for TPE makes fluorophores efficient for nonlinear microscopic studies because the ratio of the energy absorbed to the input energy flux to a specimen is high, thereby minimizing possible photodamage to specimens [39, 40]. When two-photon techniques are used to image molecular activities in living biological preparations and turbid tissues, a favorable cross section is desirable [61]. In the present study, the absolute cross section for TPE calculated for the water-soluble C_60_(OH)_46_ was approximately 1037 GM (Goeppert-Mayer unit, with 1 GM = 10^−50^cm^4^s photon^−1^) at a 760-nm excitation wavelength (fluorescein was the standard reference for the cross section [39, 40]; Fig. 1b and Tables 1 and 3); the absolute cross section calculated for the water-soluble C_60_(OH)_21_ was approximately 1230 GM, which is similar to values obtained in relevant studies [62, 63]. These absolute cross sections could facilitate the two-photon process. Moreover, the fluorescence of the water-soluble C_60_(OH)_46_ was illuminated through a two-photon process (Fig. 1b). The relative fluorescence QY was approximately 0.02 (the QY of Cy5.5 in dimethyl sulfoxide [31] served as a reference: QY_ref_ = 0.28); similarly, the absolute QY [64] was approximately 0.01, and the same QYs were derived for one-photon excitation and TPE [31]. By contrast, the water-soluble C_60_(OH)_21_ had lower relative and absolute QYs (0.06 and 0.05, respectively). In addition, this study investigated the lifetime of the fullerenols. The effects of radiative and nonradiative decay rates on QY and lifetime were calculated. The average lifetime of the water-soluble C_60_(OH)_46_ was approximately 7.797 ns, as calculated from observed lifetimes of 0.149, 1.775, and 19.679 ns; the average lifetime of the water-soluble C_60_(OH)_21_ was approximately 5.251 ns (Fig. 7 and Table 4). Therefore, the ratio of radiative to nonradiative decay rates of the water-soluble C_60_(OH)_46_ was approximately0.020 (derived from rates of approximately 2.565 × 10^6^ s^−1^ to 1.257 × 10^8^ s^−1^), whereas that of the water-soluble C_60_(OH)_21_ was approximately 0.064 (approximately 1.143 × 10^7^ s^−1^ and 1.790 × 10^8^ s^−1^; Additional file 1: Table S4). This finding is attributable to the existence of a hydroxyl group on the surface of the water-soluble fullerenol, which induced the nonradiative recombination of electron–hole pairs, leading to the inhibition of intrinsic state emission. However, hydroxylgroups at the edge of the water-soluble fullerenol may have a high occupied molecular orbital. This can be attributed to the strong orbital interaction between hydroxyl groups, which could thus increase the efficiency of intersystem crossing (rather than fluorescence generation) and generate numerous nanomaterial triplets with a high composition of hydroxyl groups; therefore, this would result in a high *Φ*_Δ_ value for the water-soluble fullerenol and induce the fullerenol to react with oxygen according to the Jablonski diagram [65]. Consequently, two-photon PDT can be effectively performed using ultralow energy in an extremely short time, thereby providing an alternative approach to killing malignant species.

**Table 3:** TPE cross section of materials (Ex, 760 nm)

Reference	Integrated emission intensity (counts)		Action cross section (ησ)
Rhodamine B	268.49		66.1
Sample	Integrated emission intensity (counts)	Relative quantum yield (η)	Absolute cross section (σ)
C_60_(OH)_46_	84.26	0.02	1037.21
C_60_(OH)_21_	299.89	0.06	1230.51

Rhodamine B was selected as the standard reference for the TPE cross section, and the relevant caluclations are presented in Materials and Methods

**Table 4:** The lifetime data and the parameter generated using a time-correlated single-photon counting technique involving a triple-exponential fitting function while monitoring the emission with 760 nm of wavelength under TPE

	3 exp fitting model: (a0*exp(a1x) + a2*exp(a3x) + a4*exp(a5x) + a6)							Lifetime1	Lifetime2	Lifetime3	Average lifetime (ns)
	a0	a1	a2	a3	a4	a5	a6				
C_60_(OH)_46_	112.448	− 6.69057	1089.64	− 0.56349	624.637	− 0.05082	− 291.897	0.149464	1.77467	19.67907	7.796924026
C_60_(OH)_21_	112.968	− 1.00006	156.714	− 0.28551	129.934	− 0.09045	− 5.90298	0.99994	3.50248	11.05533	5.250821264

The parameters were obtained from the iterative reconvolution of the decay with the instrument response function

## Conclusions

This study revealed that a water-soluble fullerenol material with a higher composition of hydroxyl groups had superior photoproperties to those of a fullerenol material with a lower composition of hydroxyl groups; the superior photoproperties can be attributed to the reduced laser exposure and materials used for treatment. Furthermore, the water-soluble fullerenol with a higher composition of hydroxyl groups exhibited high TPA, a favorable absolute cross section for TPE, and high two-photon stability. Therefore, this fullerenol has potential as a two-photon PS in two-photon PDT coupled with TPE. This property is probably due to the presence of a hydroxyl group on the surface of the water-soluble fullerenol, which caused the nonradiative recombination of electron–hole pairs, leading to the inhibition of intrinsic state emission. Moreover, hydroxyl groups at the edge of the water-soluble fullerenol may have a high occupied molecular orbital; this may be ascribed to the strong orbital interaction between the hydroxyl groups, thereby increasing intersystem crossing (rather than fluorescence generation) efficiency and generating numerous material triplets with a high composition of hydroxyl groups. Therefore, the water-soluble fullerenol would have a high *Φ*_Δ_ value and react with oxygen according to the Jablonski diagram. Consequently, two-photon PDT can be effectively performed using ultralow energy in an extremely short time. Accordingly, this efficient alternative approach to managing malignant species presents possibilities for future clinical applications.

## Supplementary information

**Additional file 1: Figure S1.** Growth curves for bacteria and water-soluble a C_60_(OH)_46_- or b C_60_(OH)_21_-treated-bacteria. **Table S1.** Stability of well-prepared water-soluble fullerenol in physiological environments. **Figure S2.** Field desorption mass spectrometry spectra of fullerenol. **Table S2.** The amount of ROS generated from by a TPE (211.2 nJ pixel^−1^, 800 scans; Ex, 760 nm) to water-soluble fullerenol (3 or 6 μg mL^−1^) was conducted in the dark and monitored. **Figure S3.** Functional characterization of synthesized water-soluble C_60_(OH)_21_. **Figure S4.** Field desorption mass spectrometry spectra of fullerenol. **Table S3.** The amount of ROS generated from by a TPE (211.2 nJ pixel^−1^, 800 scans; Ex, 760 nm) to water-soluble fullerenol-treated-*Bacillus subtilis* (*B. subtilis*; 3 or 6 μg mL^−1^) was conducted in the dark and monitored. **Figure S5.** Viability (%) was quantified according to the determined viable count of material-treated *B. subtilis* through a CFU assay conducted using short excitation with a TPE power of 211.2 nJ pixel^−1^ with 800 scans (approximately 3.2621 s of total effective exposure time; Ex, 760 nm). **Figure S6.** The number of surviving material-treated-bacteria was determined by CFU counting assay. **Figure S7.** The temperature dependence of material as a function of irradiaiton time with a TPE power of 211.2 nJ pixel^−1^ (Ex, 760 nm). **Table S4.** The radiative and non-radiative decay rates of water-soluble fullerenol.

## Data Availability

All datasets are presented in the main paper.

## Abbreviations

PS: Photosensitizers
QY: Quantum yield
PDT: Photodynamic therapy
UV–vis: Ultraviolet–visible
ROS: Reactive oxygen species
*ψ*_Δ_: Singlet oxygen QY
NIR: Near-infrared
TPE: Two-photon excitation
*E. coli*: *Escherichia coli*
HR-TEM: High-resolution transmission electron microscopy
DLS: Dynamic light scattering
FTIR: Fourier-transform infrared
XPS: X-ray photoelectron spectroscopy
FD: Field desorption
CFU: Colony forming unit
TSPP: *Meso*-tetra(4-sulfonatophenyl)porphine dihydrochloride
PL: Photoluminescence
DMSO: Dimethyl sulfoxide
OPE: One-photon excitation
TPA: Two-photon absorption
TPL: Two-photon luminescence
ti-sa: Titanium-sapphire
*NA*: Numerical aperture
FLIM: Fluorescence lifetime imaging microscopy
^1^O_2_: Singlet oxygen
SOSG: Singlet Oxygen Sensor Green
*t*-MVP: Trans-1-(2′-methoxyvinyl)pyrene
O_2_^**.**−^: Superoxide radical anion
XTT: 2, 3-Bis (2-methoxy-4-nitro-5-sulfophenyl)-2H-tetrazolium-5-carboxanilide
GSH: Glutathione, *γ*-l-glutamyl-l-cysteinyl-glycine
S_1_: Singlet
T_1_: Triplet
*B. subtilis*: *Bacillus subtilis*
GM: Goeppert-Mayer
