# Supplementary material for: Water-Soluble Fullerenol with Hydroxyl Group Dependence for Efficient Two-Photon Excited Photodynamic Inactivation of Infectious Microbes
Source: Nanoscale Res Lett. 2020 May 6;15:99. doi: 10.1186/s11671-020-03329-6 (PMC7203358; doi:10.1186/s11671-020-03329-6)
Supplement: Supplementary file 1 — Additional file 1: Figure S1. Growth curves for bacteria and water-soluble a C60(OH)46- or b C60(OH)21-treated-bacteria. Table S1. Stability of well-prepared water-soluble fullerenol in physiological environments. Figure S2. Field desorption mass spectrometry spectra of fullerenol. Table S2. The amount of ROS generated from by a TPE (211.2 nJ pixel−1, 800 scans; Ex, 760 nm) to water-soluble fullerenol (3 or 6 μg mL−1) was conducted in the dark and monitored. Figure S3. Functional characterization of synthesized water-soluble C60(OH)21. Figure S4. Field desorption mass spectrometry spectra of fullerenol. Table S3. The amount of ROS generated from by a TPE (211.2 nJ pixel−1, 800 scans; Ex, 760 nm) to water-soluble fullerenol-treated-Bacillus subtilis (B. subtilis; 3 or 6 μg mL−1) was conducted in the dark and monitored. Figure S5. Viability (%) was quantified according to the determined viable count of material-treated B. subtilis through a CFU assay conducted using short excitation with a TPE power of 211.2 nJ pixel−1 with 800 scans (approximately 3.2621 s of total effective exposure time; Ex, 760 nm). Figure S6. The number of surviving material-treated-bacteria was determined by CFU counting assay. Figure S7. The temperature dependence of material as a function of irradiaiton time with a TPE power of 211.2 nJ pixel−1 (Ex, 760 nm). Table S4. The radiative and non-radiative decay rates of water-soluble fullerenol. [file 11671_2020_3329_MOESM1_ESM.pdf]

Supplementary Information

Additional file 1

Water-Soluble Fullerenol with Hydroxyl Group Dependence for Efficient Two-Photon Excited Photodynamic Inactivation of Infectious Microbes

Wen-Shuo Kuo[1,2], Jiu-Yao Wang[2,3,4], Chia-Yuan Chang[5], Jui-Chang Liu[2,3], Yu-Ting Shao[2,4], Yen-Sung Lin[6,7], Edmund Cheung So[8,9,10*], and Ping-Ching Wu[11*]*

[1]School of Chemistry and Materials Science, Nanjing University of Information Science and Technology, Nanjing 210044, Jiangsu, China.

[2]Allergy & Clinical Immunology Research Center, National Cheng Kung University Hospital, College of Medicine, National Cheng Kung University, Tainan 701, Taiwan (R.O.C.)

[3]Department of Biochemistry and Molecular Biology, National Cheng Kung University Hospital, College of Medicine, National Cheng Kung University, Tainan 701, Taiwan (R.O.C.)

[4]Department of Microbiology & Immunology, National Cheng Kung University Hospital, College of Medicine, National Cheng Kung University, Tainan 701, Taiwan (R.O.C.)

[5]Department of Mechanical Engineering, National Cheng Kung University, Tainan 701, Taiwan (R.O.C.)

[6]Division of Pulmonary and Critical Care Medicine, An Nan Hospital, China Medical University, Tainan 709, Taiwan (R.O.C.)

[7]Department of Nursing, Chung Hwa University of Medical Technology, Tainan 717, Taiwan (R.O.C.)

[8]Department of Anesthesia & Medicine Research, An Nan Hospital, China Medical University, Tainan 709, Taiwan (R.O.C.)

[9]Department of Anesthesia, China Medical University, Taichung 404, Taiwan (R.O.C.)

[10]Graduate Institute of Medical Sciences, Chang Jung Christian University, Tainan 711, Taiwan (R.O.C.)

[11]Department of Biomedical Engineering, National Cheng Kung University, Tainan 701, Taiwan (R.O.C.)

*To whom correspondence should be addressed. E-mail: chestlin@gmail.com (YSL); edmundsotw@gmail.com (ECS); wbcxyz@bme.ncku.edu.tw (PCW)

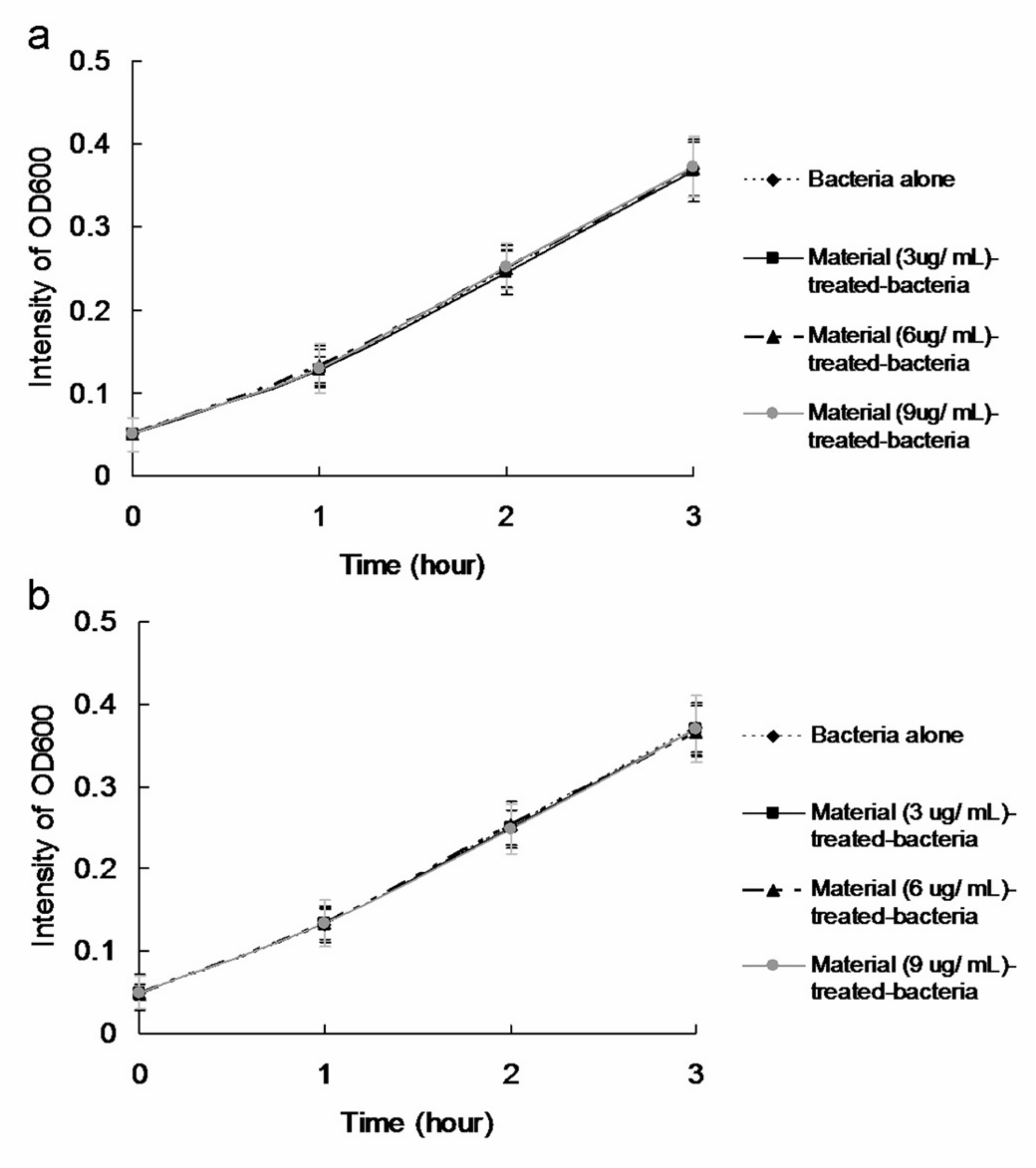

Fig. S1 Growth curves for bacteria and water-soluble **a** $C_{60}(OH)_{46}$- or **b** $C_{60}(OH)_{21}$-treated-bacteria. Delivered dose: $OD_{600} \sim 0.05$ of *E. coli* and 0-9 μg mL^{-1} water-soluble fullerenol. Data are means ± SD (*n*=6).

Table S1 Stability of well-prepared water-soluble fullerenol in physiological environments.

	$C_{60}(OH)_{46}$		$C_{60}(OH)_{21}$	
	Newly prepared	Prepared and stayed for 3 months	New prepared	Prepared and stayed for 3 months
pH 7 aqueous solution	130	129	131	130
1X PBS	129	131	131	131
Culture medium of *E. coli*	131	130	132	132

To subtract the approximately m/z value (720) of C_{60} from that (1512) and then divide by approximately 17 (m/z value of hydroxyl group), resulting in approximately 46. Consequentially, the number of hydroxyl groups was confirmed to be 46 based on the results provided by the field desorption mass spectrometer (**Fig. S2**).

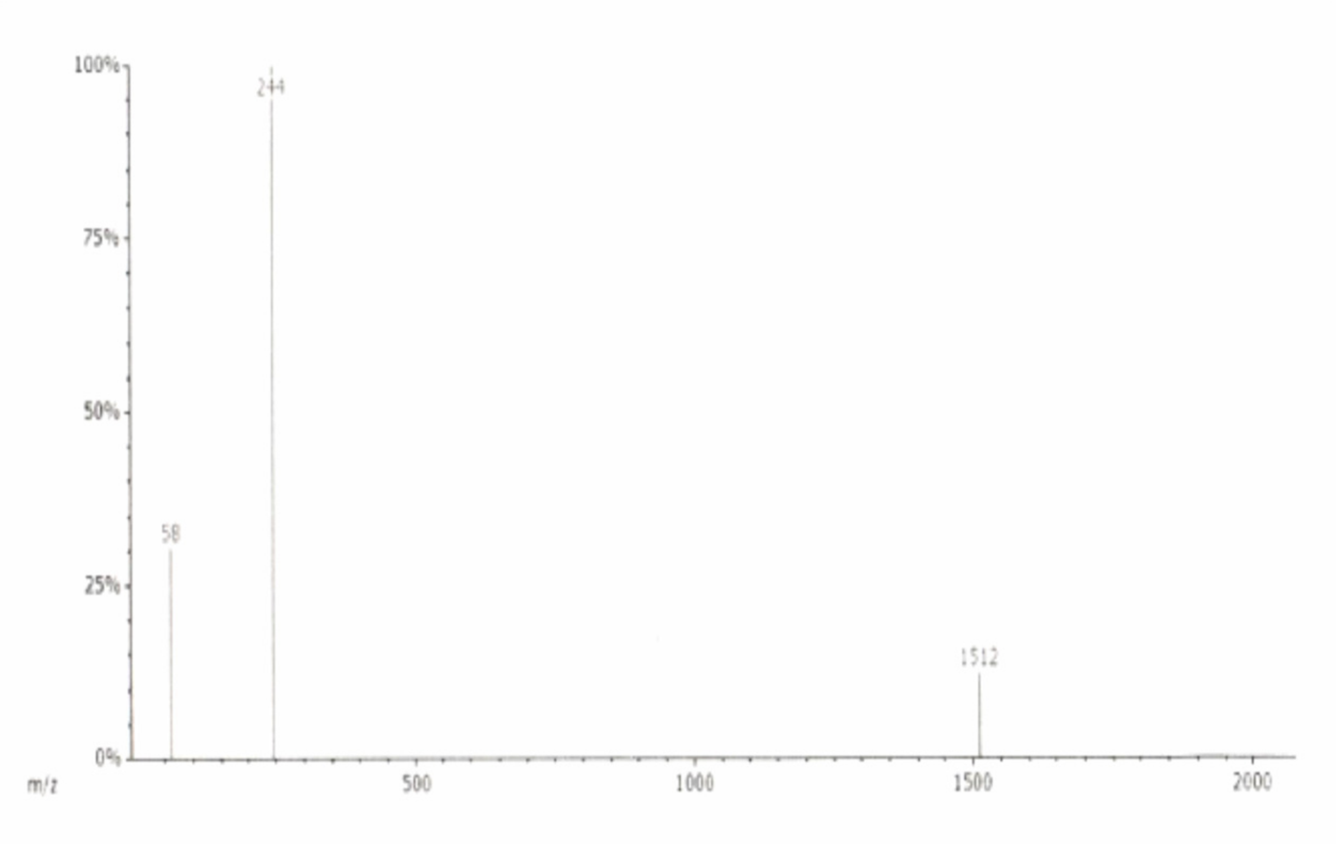

Fig. S2 Field desorption mass spectrometry spectra of fullerenol.

Table S2 The amount of ROS generated [1-12] from by a TPE (211.2 nJ pixel^{-1}, 800 scans; Ex: 760 nm) to water-soluble fullerenol (3 or 6 μg mL^{-1}) was conducted in the dark and monitored. Data are means ± SD (n=6).

	$^1O_2$ (by SOSG)[c]						
	Negative control[ac]	ROS neutralization[abc]	Positive control[cd]	$C_{60}(OH)_{46}$	ROS neutralization[bc]	$C_{60}(OH)_{21}$	ROS neutralization[b]
3 μg mL^{-1}	234±12	233±11	2802±131	2637±124	232±10	2303±109	232±11
6 μg mL^{-1}	233±12	233±13	2816±135	2755±126	233±11	2398±112	233±11

	$^1O_2$ (by *t*-MVP)[e]						
	Negative control[ae]	ROS neutralization[abe]	Positive control[de]	$C_{60}(OH)_{46}$	ROS neutralization[be]	$C_{60}(OH)_{21}$	ROS neutralization[be]
3 μg mL^{-1}	340±21	342±21	9301±228	9049±211	341±21	8627±181	340±20
6 μg mL^{-1}	341±21	341±22	9326±232	9151±218	342±20	8743±190	341±20

	$O_2\bullet^-$ (by XTT)[f]						
	Negative control[af]	ROS neutralization[abf]	Positive control[df]	$C_{60}(OH)_{46}$	ROS neutralization[bf]	$C_{60}(OH)_{21}$	ROS neutralization[bf]
3 μg mL^{-1}	0	0	1.95±0.13	1.88±0.10	0.02±0.01	1.76±0.07	0.02±0.01
6 μg mL^{-1}	0	0	1.97±0.16	1.93±0.12	0.03±0.01	1.84±0.09	0.02±0.01

	$O_2\bullet^-$ (by GSH)[g]						
	Negative control[ag]	ROS neutralization[abg]	Positive control[dg]	$C_{60}(OH)_{46}$	ROS neutralization[bg]	$C_{60}(OH)_{21}$	ROS neutralization[bg]
3 μg mL^{-1}	0	0	99.1±4.5%	90.2±4.1%	0.2±0.1%	82.7±3.6%	0.2±0.1%
6 μg mL^{-1}	0	0	99.5±4.3%	94.5±4.2%	0.2±0.1%	85.8±3.8%	0.3±0.1%

[a]Negative control: only treat reagent and laser radiation without material (0 μg mL^{-1}).

[b]ROS neutralization: with the treatments of nanomaterial, the laser irradiation and 30 ppm of antioxidant α-Tocopherol/methyl linoleate.

[c]SOSG reagent (Ex/Em: 488/525 nm) has a specific reactivity to generate fluorescence recorded by a PL spectrometer.

[d]Positive control: the treatment of 50 μM *tert*-butyl hydroperoxide (TBHP) and laser irradiation.

[e]*t*-MVP (Ex/Em: 352/465 nm) can react with $^1O_2$, forming a dioxetane intermediate that generates fluorescence upon decomposition to 1-pyrenecarboxaldehyde, and monitored by a PL spectrometer.

[f]XTT would interact with $O_2^{\cdot-}$ and produce the XTT-formazan generating strong

absorption (470 nm in wavelength).

[g]GSH containing a thiol-tripeptide can prevent damages to cellular or bacterial components caused by stress of oxidation. Thiol group from GSH can be oxidized to disulfide bond converting GSH to glutathione disulfide. GSH oxidation was used to determine the generated $O_2^{\cdot -}$. Loss of GSH (%) = (absorbance difference between of sample and negative control / absorbance of negative control) × 100 %.

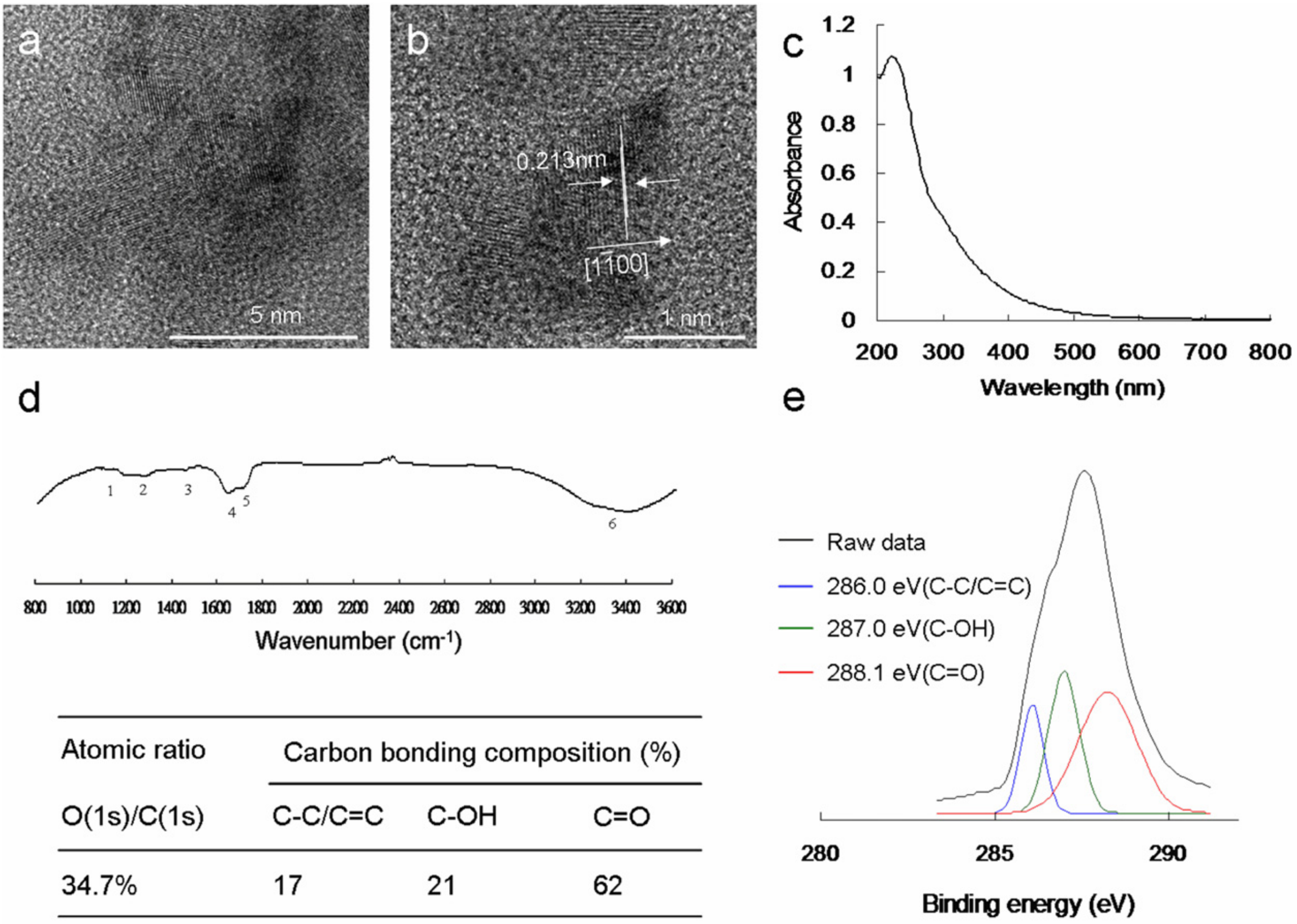

Fig. S3 Functional characterization of synthesized water-soluble $C_{60}(OH)_{21}$. **a** Low-magnified TEM image and **b** HR-TEM image of water-soluble $C_{60}(OH)_{21}$, illustrating that the materials had $\{1\bar{1}00\}$ lattice planes and a mean size of 1.13 ± 0.04 nm with a *d*-spacing of 0.213 nm. **c** UV–vis spectrum revealing nanomaterial peaks at approximately 216 and 310 nm, which were attributed to the π–π* transition of aromatic C=C bonds and n–π* transitions of the C=O shoulder, respectively. **d** FTIR spectra revealing characteristic bands of nanomaterials: C–O stretching band at approximately 1102 cm^{-1} (band 1); phenolic C–OH stretching band at approximately 1255 cm^{-1} (band 2); tertiary alcoholic C=O stretching band at approximately 1429 cm^{-1} (band 3); C=C ring stretching band at approximately 1667 cm^{-1} (band 4); C=O ring stretching band at approximately 1730 cm^{-1} (band 5); and C–H, intermolecular hydrogen bonded, and carboxylate O–H stretching band at approximately 3347 cm^{-1} (band 6). **e** Deconvoluted C(1s) XPS spectra and fitted peaks obtained using Gaussian function: nonoxygenated ring (C–C/C=C, 286.0 eV), C–O bond (287.0 eV), and C=O bond (288.1 eV). The atomic ratio and bonding composition of the fullerenol are shown as summarized in the table. The O(1s)/C(1s) atomic ratio was 34.7%.

To subtract the approximately m/z value (720) of C_{60} from that (1086) and then divide by approximately 17 (m/z value of hydroxyl group), resulting in approximately 21. Consequentially, the number of hydroxyl groups was confirmed to be 21 based on the results provided by the field desorption mass spectrometer (**Fig. S4**).

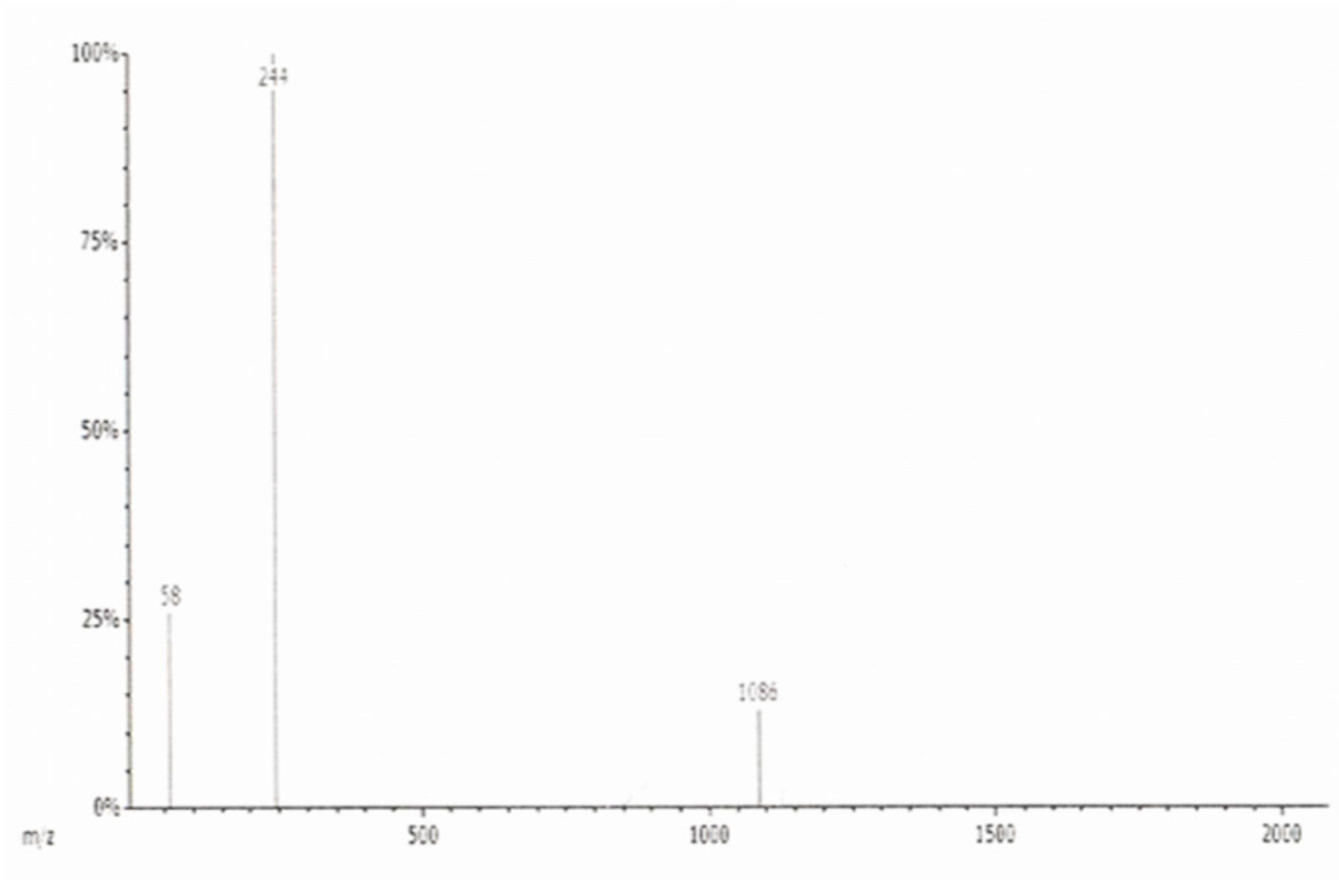

Fig. S4 Field desorption mass spectrometry spectra of fullerenol.

Table S3 The amount of ROS generated [1-12] from by a TPE (211.2 nJ pixel^{-1}, 800 scans; Ex: 760 nm) to water-soluble fullerenol-treated-*Bacillus subtilis* (*B. subtilis*; 3 or 6 μg mL^{-1}) was conducted in the dark and monitored. Data are means ± SD (*n*=6).

$^1O_2$ (by SOSG)[c]							
	Negative control[ac]	ROS neutralization[abc]	Positive control[cd]	$C_{60}(OH)_{46}$	ROS neutralization[bc]	$C_{60}(OH)_{21}$	ROS neutralization[bc]
3 μg mL^{-1}	231±11	230±10	2784±129	2537±102	231±10	2224±101	232±10
6 μg mL^{-1}	230±9	231±12	2813±135	2592±113	230±11	2306±109	231±10

$^1O_2$ (by *t*-MVP)[e]							
	Negative control[ae]	ROS neutralization[abe]	Positive control[de]	$C_{60}(OH)_{46}$	ROS neutralization[be]	$C_{60}(OH)_{21}$	ROS neutralization[be]
3 μg mL^{-1}	338±20	339±21	9319±234	8882±197	340±23	8566±168	340±19
6 μg mL^{-1}	340±19	340±22	9336±238	8950±204	341±22	8623±175	341±18

$O_2\bullet^-$ (by XTT)[f]							
	Negative control[af]	ROS neutralization[abf]	Positive control[df]	$C_{60}(OH)_{46}$	ROS neutralization[bf]	$C_{60}(OH)_{21}$	ROS neutralization[bf]
3 μg mL^{-1}	0	0	1.96±0.17	1.88±0.11	0.02±0.02	1.78±0.10	0.03±0.01
6 μg mL^{-1}	0	0	1.99±0.19	1.91±0.13	0.02±0.01	1.82±0.12	0.02±0.01

$O_2\bullet^-$ (by GSH)[g]							
	Negative control[ag]	ROS neutralization[abg]	Positive control[dg]	$C_{60}(OH)_{46}$	ROS neutralization[bg]	$C_{60}(OH)_{21}$	ROS neutralization[bg]
3 μg mL^{-1}	0	0	99.3±4.1%	84.1±3.0%	0.2±0.1%	76.2±2.8%	0.3±0.1%
6 μg mL^{-1}	0	0	99.8±4.5%	87.5±3.3%	0.2±0.1%	82.1±3.4%	0.2±0.1%

[a]Negative control: only treat reagent and laser radiation without material (0 μg mL^{-1}).

[b]ROS neutralization: with the treatments of nanomaterial, the laser irradiation and 30 ppm of antioxidant α-Tocopherol/methyl linoleate.

[c]SOSG reagent (Ex/Em: 488/525 nm) has a specific reactivity to generate fluorescence recorded by a PL spectrometer.

[d]Positive control: the treatment of 50 μM TBHP and laser irradiation.

[e]*t*-MVP (Ex/Em: 352/465 nm) can react with $^1O_2$, forming a dioxetane intermediate that generates fluorescence upon decomposition to 1-pyrenecarboxaldehyde, and monitored by a PL spectrometer.

[f]XTT would interact with $O_2^{\cdot -}$ and produce the XTT-formazan generating strong absorption (470 nm in wavelength).

[g]GSH containing a thiol-tripeptide can prevent damages to cellular or bacterial

components caused by stress of oxidation. Thiol group from GSH can be oxidized to disulfide bond converting GSH to glutathione disulfide. GSH oxidation was used to determine the generated $O_2^{\cdot -}$. Loss of GSH (%) = (absorbance difference between of sample and negative control / absorbance of negative control) × 100 %.

Culture of *B. subtilis*

B. subtilis (ATCC 14593) were grown in nutrient agar (DIFCO 0001, BD, Franklin Lakes, NJ, USA) and incubated at 30 °C: beef extract 3.0 g, peptone 5.0 g, bacto agar 15.0 g, and distilled water 1.0 liter (pH= 7.0).

Fig. S5 shows that without photoexcitation treatment there was no antimicrobial effect on *Bacillus subtilis* (*B. subtilis*) in the two panels, as well as bacteria alone with photoexcitation. For the panel on water-soluble $C_{60}(OH)_{21}$-treated-bacteria with photoexcitation treatment, bacterial viability sharply decreased with delivered doses (3 μg mL^{-1}), killing nearly 87%, corresponding to an approximately 0.770 log_{10} reduction after TPE (**Fig. S5a,b**). By contrast, the bacterial viability observed for the panel treated with water-soluble $C_{60}(OH)_{46}$ was approximately 0 (100% elimination efficiency, corresponding to an approximately 7.875 log_{10} reduction). When the dose was increased (6 μg mL^{-1}), a complete bactericidal effect was observed for both materials (**Fig. S5c,d**).

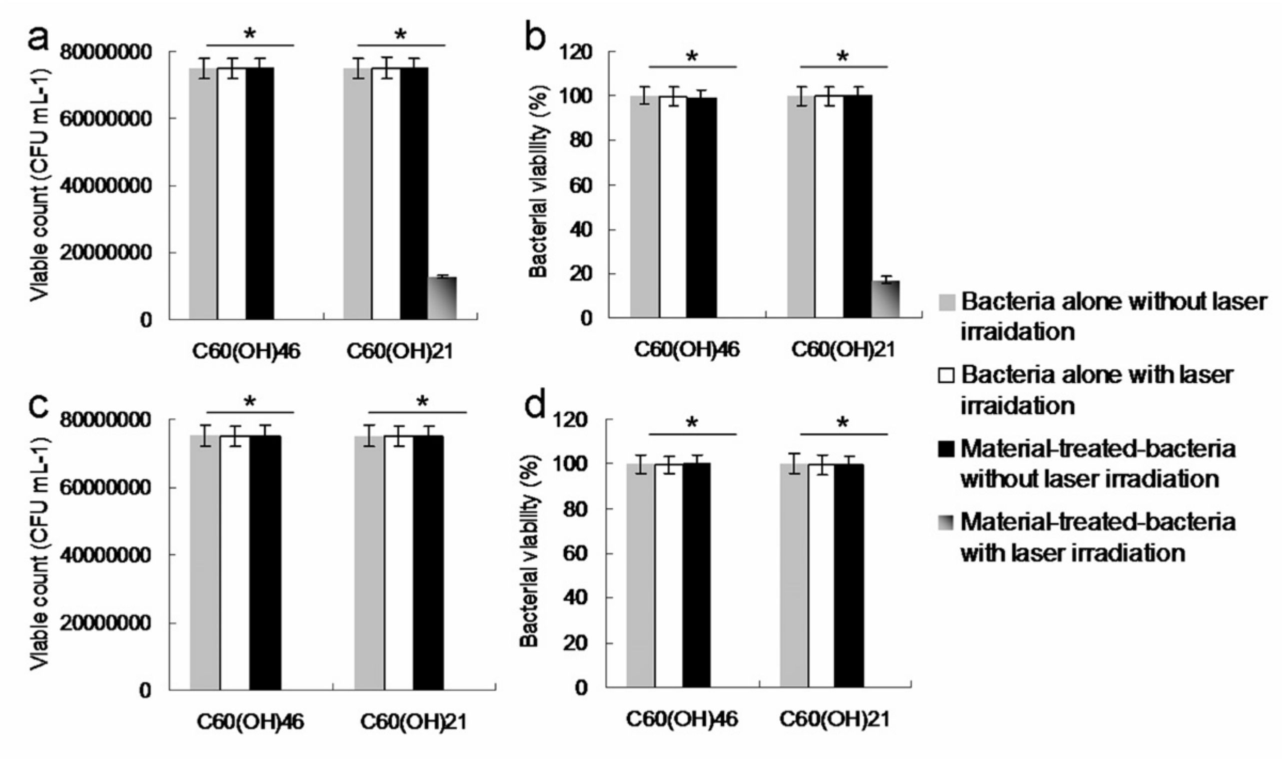

Fig. S5 Viability (%) was quantified according to the determined viable count of material-treated *B. subtilis* through a CFU assay conducted using short excitation with a TPE power of 211.2 nJ $pixel^{-1}$ with 800 scans (approximately 3.2621 s of total effective exposure time; Ex: 760 nm) to deliver a dose of **a,b** 3 or **c,d** 6 μg mL^{-1}. Delivered dose: OD_{600}–0.05 of *B. subtilis.* Data are presented as means ± SD (n=6). For $C_{60}(OH)_{46}$- and $C_{60}(OH)_{21}$-treated *B. subtilis* with photoexcitation, **a** $p < 0.001$ and $p = 0.420$, **b** $p < 0.001$ and $p = 0.424$, **c** $p < 0.001$ and $p < 0.001$, and **d** $p < 0.001$ and $p < 0.001$. *p value obtained by Student's t test.

<u>Culture of *B. subtilis*</u>

B. subtilis (ATCC 14593) were grown in nutrient agar (DIFCO 0001, BD, Franklin Lakes, NJ, USA) and incubated at 30 °C: beef extract 3.0 g, peptone 5.0 g, bacto agar 15.0 g, and distilled water 1.0 liter (pH= 7.0).

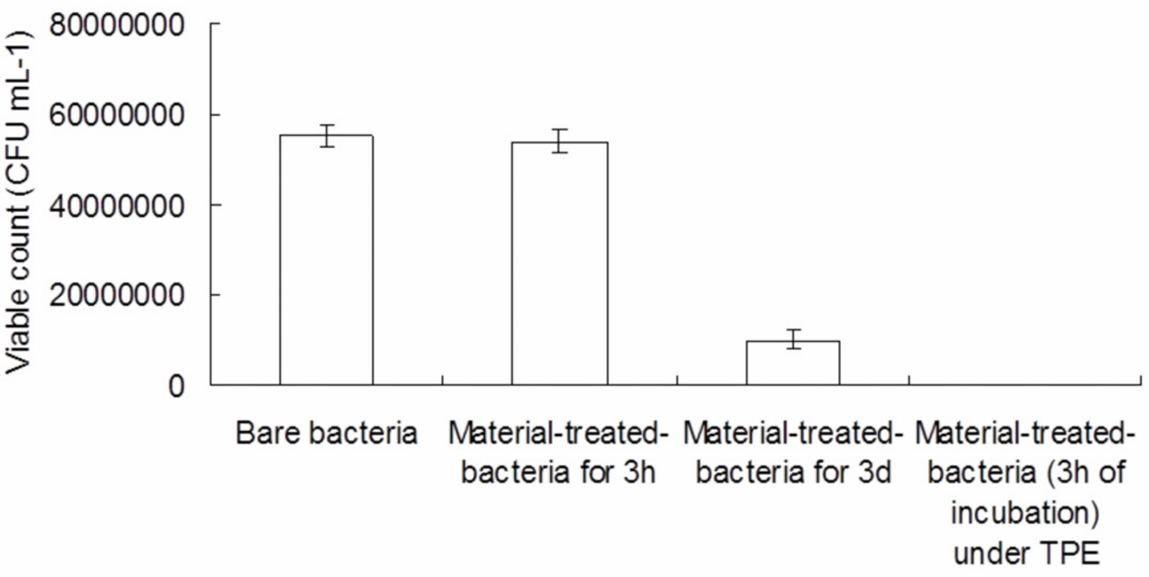

Fig. S6 The number of surviving material-treated-bacteria was determined by CFU counting assay, corresponding to **Fig. 5f**, which is expressed as the percentage (%) for bacteria that corresponds to the unit of CFU mL^{-1}. Delivered dose: OD_{600} ~ 0.05 of *E. coli* and 3 μg mL^{-1} water-soluble fullerenol $C_{60}(OH)_{46}$. Data are means ± SD (n=6).

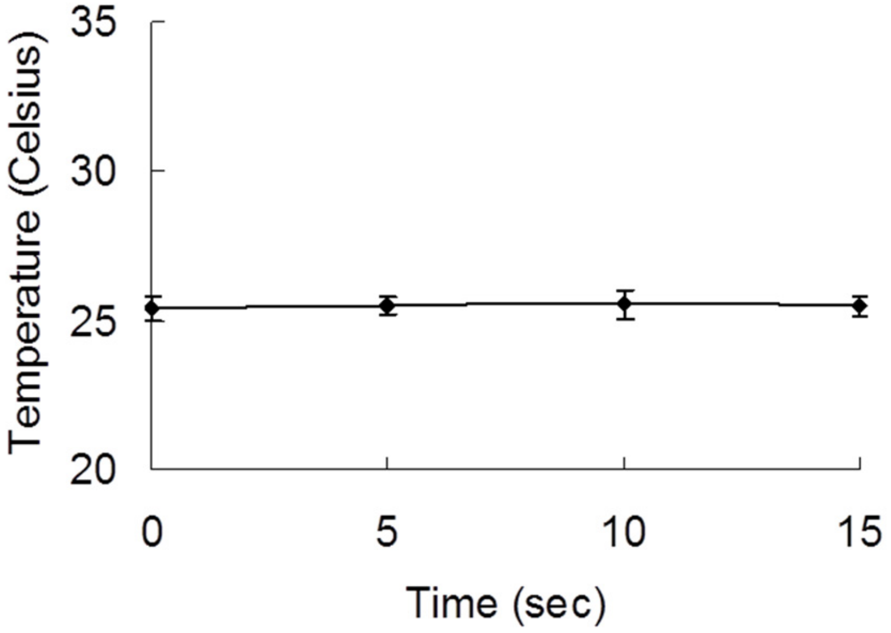

Fig. S7 The temperature dependence of material as a function of irradiaiton time with a TPE power of 211.2 nJ pixel^{-1} (Ex: 760 nm). Delivered dose: OD_{600} ~ 0.05 of *E. coli* and 3 μg mL^{-1} water-soluble fullerenol $C_{60}(OH)_{46}$. Data are means ± SD (*n*=6).

Table S4 The radiative and non-radiative decay rates of water-soluble fullerenol.[a]

	Radiative decay rate (s^{-1})	Non-radiative decay rate (s^{-1})	Ratio of the rates of radiative to non-radiative decay
$C_{60}(OH)_{46}$	2.5651×10^{6}	1.2569×10^{8}	0.0204
$C_{60}(OH)_{21}$	1.1427×10^{7}	1.7902×10^{8}	0.0638

[a]The calculation was based on Equations 6 and 7 in Experimental section.

Reference

1. Chang WT, Chen SJ, Chang CY, Liu YH, Chen CH, Yang CH Chou LCS, Chang JC, Cheng LC, Kuo WS. Wang JY (2015) Effect of size-dependence photodestructuve efficacy by gold nanomaterials with multiphoton laser. ACS Appl Mater Interfaces 7: 17318–17329.

2. Kuo WS, Hsu CLL, Chen HH, Chang CY, Kao HF, Chou LCS, Chen YC, Chen SJ, Chang WT, Tseng SW, Wang JY, Pu YC (2016) Graphene quantum dots conjugated with polymers for two-photon properties under two-photon excitation. Nanoscale 8:16874–16880.

3. Wu PC, Wang JY, Wang WL, Chang CY, Huang CH, Yang KL, Chang JC, Hsu CLL, Chen SY, Chou TM, Kuo WS (2018) Efficient two-photon luminescence for cellular imaging using biocompatible nitrogen-doped graphene quantum dots conjugated with polymers. Nanoscale 10:109–117.

4. Kinen MM, Kamal-Eldin A, Lampi AM, Hopia A (2000) Effects of α- and γ-tocopherols on formation of hydroperoxides and two decomposition products from methyl linoleate. J Am Oil Chem Soc 77:801–806.

5. Possel H, Noack H, Augustin W, Keilhoff G, Wolf G (1997) An oxidant, *tert*-butyl hydroperoxide (TBHP), to serve as a positive control. FEBS Lett 416:175–178.

6. Thompson A, Lever JR, Canella KA, Miura KA, Posner GH, Seliger HH (1986) Chemiluminescence mechanism and quantum yield of synthetic vinylpyrene analogues of benzo[*a*]pyrene-7,8-dihydrodiol. J Am Chem Soc 108:4498–4504.

7. Feng X, Li X, Li Z, Liu Y (2016) Size-dependent two-photon absorption in circular graphene quantum dots. Opt Express 24:2877–2884.

8. Kuo WS, Chang CN, Chang YT, Yeh CS (2009) Antimicrobial gold nanorods dual-modality photodynamic therpay inactivation and hyperthermia. Chem Commun 4853−4855.

9. Sharma P, Jha AB, Dubey RS, Pessarakli M (2012) Reactive oxygen species, oxidative damage, and antioxidative defense mechanism in plants under stressful conditions. J Bot 2012:1–26.

10. Kuo WS, Chang CY, Chen HH, Hsu CLL, Wang JY, Kao HF, Chou LCS, Chen YC, Chen SJ, Chang WT, Tseng SW, Wu PC, Pu YC (2016) Two-photon photoexcited photodynamic therapy and contrast agent with antimicrobial graphene quantum dots. ACS Appl Mater Interfaces 8:30467–30474.

11. Ellman GL (1959) Tissue sulfhydryl gropus. Arch Biochem Biophys 82:70–77.

12. Carmel-Hare O, Storz G (2000) Roles of the glutathione- and thioredoxin-dependent reduction systems in the *Escherichia coli* and *Saccaromyces cerevisiae* responses to oxidative stress. Annu Rev Microbiol 54:439–461.
